# Exposures to temperature beyond threshold disproportionately reduce vegetation growth in the northern hemisphere

**DOI:** 10.1093/nsr/nwy158

**Published:** 2018-12-22

**Authors:** Xiuchen Wu, Weichao Guo, Hongyan Liu, Xiaoyan Li, Changhui Peng, Craig D Allen, Cicheng Zhang, Pei Wang, Tingting Pei, Yujun Ma, Yuhong Tian, Zhaoliang Song, Wenquan Zhu, Yang Wang, Zongshan Li, Deliang Chen

**Affiliations:** 1 State Key Laboratory of Earth Surface Processes and Resource Ecology, Beijing Normal University, Beijing 100875; 2 Faculty of Geographical Science, Beijing Normal University, Beijing 100875, China; 3 College of Urban and Environmental Science, Peking University, Beijing 100871, China; 4 Institute of Environment Sciences, University of Quebec at Montreal, Quebec G1K 9H7, Canada; 5 US Geological Survey, Fort Collins Science Center, Jemez Mountains Field Station, Los Alamos, NM, 87544, USA; 6 Institute of the Surface-Earth System Science Research, Tianjin University, Tianjin 300072, China; 7 State Key Laboratory of Urban and Regional Ecology, Research Center for Eco-Environmental Sciences, Chinese Academy of Sciences, Beijing 100085, China; 8 Regional Climate Group, Department of Earth Sciences, University of Gothenburg, Gothenburg 460, Sweden

**Keywords:** temperature exposure, vegetation growth, extremely high temperature, non-linear response, temperate and boreal northern hemisphere

## Abstract

In recent decades, terrestrial vegetation in the northern hemisphere (NH) has been exposed to warming and more extremely high temperatures. However, the consequences of these changes for terrestrial vegetation growth remain poorly quantified and understood. By examining a satellite-based vegetation index, tree-ring measurements and land-surface model simulations, we discovered a consistent convex pattern in the responses of vegetation growth to temperature exposure (TE) for forest, shrub and grass in both the temperate (30°−50° N) and boreal (50°−70° N) NH during the period of 1982−2012. The response of vegetation growth to TE for the three vegetation types in both the temperate and boreal NH increased convergently with increasing temperature, until vegetation type-dependent temperature thresholds were reached. A TE beyond these temperature thresholds resulted in disproportionately weak positive or even strong negative responses. Vegetation growth in the boreal NH was more vulnerable to extremely high-temperature events than vegetation growth in the temporal NH. The non-linear responses discovered here provide new insights into the dynamics of northern terrestrial ecosystems in a warmer world.

## INTRODUCTION

Climate warming in recent decades has resulted in a shift to a warmer temperature distribution in the extra-tropical northern hemisphere (NH), which has led to rapid, but spatially variable, increases in both the frequency and the magnitude of extremely high temperature (EHT) [1,2]. Such changes raise a critical, but often overlooked, question: how does NH vegetation growth respond to a warmer temperature distribution, other than just changes in the mean climatic state, and in particular what is the role of a markedly increased EHT [3]?

The impacts of mean warmer temperature on vegetation growth have been extensively investigated at a regional/global scale [4]. Recent evidence has pointed to a weakening interannual correlation between mean warmer temperature and vegetation growth in the NH over the past three decades [5]. Together with the widely documented ‘northern greening’ effect [6,7], this has pointed to non-linear features of the response of vegetation growth to temperature variations. The development and growth of vegetation in a stepwise manner (i.e. spanning different phenophases) depends disproportionately on the threshold-based accumulation of daily temperature and the interactions with other factors, including solar radiation, water, nutrition limitations and heat stress.

So far, the non-linear effects of temperature exposure (TE), which is defined here as the accumulated daily temperature for days on which vegetation is exposed to a specific temperature range, such as 0–1°C, 1–2°C, etc., during the growing season) within different temperature ranges on terrestrial vegetation growth have not been well quantified. A few studies have shown that the exposure of vegetation to even a short-term EHT event can result in considerable regional terrestrial growth failure [8]. More seriously, future climate scenarios consistently predict a continuous shift to warmer temperature distributions and a further intensification of EHT events [9–12]. Thus, a comprehensive understanding of the ecological responses of NH vegetation growth to TE within different temperature ranges is crucial for predicting terrestrial vegetation growth and the consequences for biogeochemical cycles and biophysical climate feedbacks in the future.

In this study, we aimed to quantify the responses of northern vegetation growth to TE within different temperature ranges, using multiple data streams, including the satellite-derived Normalized Difference Vegetation Index (NDVI), tree-ring index (TRI) and land-surface model simulations of net primary productivity (NPP).

## RESULTS

### Divergent effects of accumulated TE above and below the EHT threshold on vegetation growth

**Figure 1.**
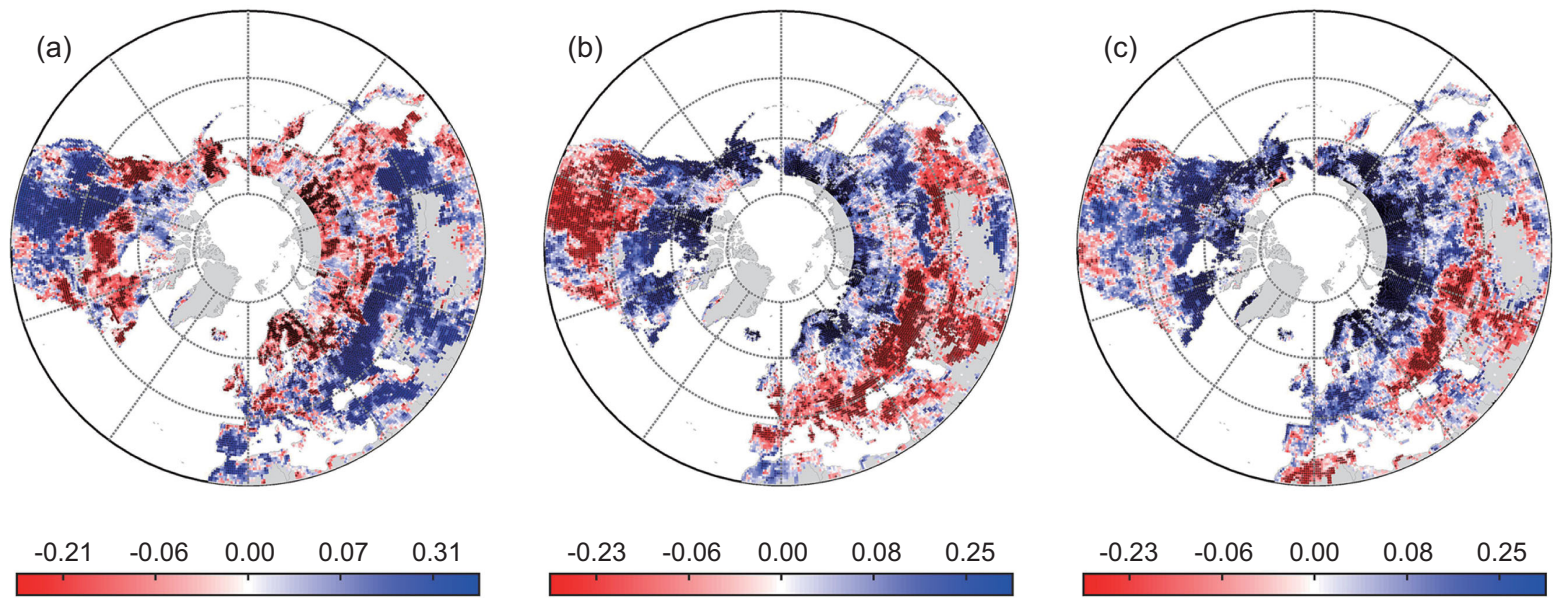
Spatial patterns of the standard regression coefficients between the interannual variations of the mean growing season (April–October) Normalized Difference Vegetation Index (NDVI_GS_) and climate. A ridge regression was performed between NDVI_GS_ and total growing season precipitation (a), accumulated temperature exposure above (b, TE_H_) and below (c, TE_L_) the 95th percentile of the daily temperature distribution for the growing seasons during the period 1982–2012. Stratified regions are statistically significant at *P* < 0.05. Regions with multi-year mean NDVI values <0.1 during 1982–2012 were discarded from our analyses (blank regions).

First, we investigated the spatial pattern in the effects of accumulated TE above (TE_H_) and below (TE_L_) the EHT threshold on vegetation growth over the temperate and boreal NH. The 95th percentile of the daily temperature distribution in growing seasons during 1982−2012 was identified as the EHT threshold for each grid. We then calculated the accumulated TE_H_ and TE_L_ during the growing season for each grid and each year during the period of 1982−2012. The accumulated growing season (defined as April−October in this study) TE_H_ increased in most of the temperate and boreal NH during 1982−2012, with the most prominent increasing trends observed in the temperate NH (Supplementary Fig. 1a, available as Supplementary Data at *NSR* online). In contrast, the accumulated growing season TE_L_ decreased during 1982−2012 (Supplementary Fig. 1b, available as Supplementary Data at *NSR* online). The ridge regression between the mean growing season NDVI (NDVI_GS_) and total growing season precipitation, TE_H_, TE_L_, mean growing season solar radiation and mean growing season temperature were statistically significant (*P* < 0.05) in ∼47% of the temperate and boreal NH, with a mean goodness of fit value (*R^2^*) of ∼0.39 (ranging from 0.27 to 0.83) (Supplementary Fig. 2, available as Supplementary Data at *NSR* online). There shows a general reduction in the Akaike information criterion (AIC) when introducing TE_H_ and TE_L_ into the ridge regression model (i.e. the second model), with a mean reduction of AIC of 2.3 ± 1.5 in our study region than the first model, despite the spatial difference. The ridge regression between the NDVI_GS_ and total growing season precipitation, TE_H_ and TE_L_ during 1982−2012 was statistically significant (*P* < 0.05) in ∼44% of the temperate and boreal NH, with a mean *R^2^* value of ∼0.36 (ranging from 0.24 to 0.84) (Supplementary Fig. 3, available as Supplementary Data at *NSR* online). These results showed that total growing season precipitation and the accumulated TE_H_ and TE_L_ together could explain a large portion of the interannual variations in the NDVI_GS_ in both the temperate and the boreal NH. We observed generally positive and negative relationships between the NDVI_GS_ and total growing season precipitation (∼68%) and TE_H_ (∼61%), with significant (*P* < 0.05) relationships in ∼35 and ∼25% of the temperate NH, respectively. Interestingly, we discovered that the accumulated TE_H_ and TE_L_ had divergent effects on the NDVI_GS_ (Fig. 1b and c). The TE_H_ exerted a more pervasive (∼61%) negative effect on the NDVI_GS_ in the temperate NH, especially in central USA and southern Eurasia, than the TE_L_ (∼48%). In boreal NH, there generally shows much weaker positive response of NDVI_GS_ to TE_H_ than TE_L_, with ∼33 and ∼47% of boreal NH with significantly (*P* < 0.05) positive response coefficients, respectively (Fig. 1b and c). Consistent patterns in the responses of NDVI_GS_ to total growing season precipitation and TE_H_ and TE_L_ were also obtained from the ridge regression analysis with different EHT definitions (90th and 99th percentiles, Supplementary Figs 4 and 5, available as Supplementary Data at *NSR* online), as well as from results of multivariate linear regression (Supplementary Fig. 6, available as Supplementary Data at *NSR* online).

**Figure 2.**
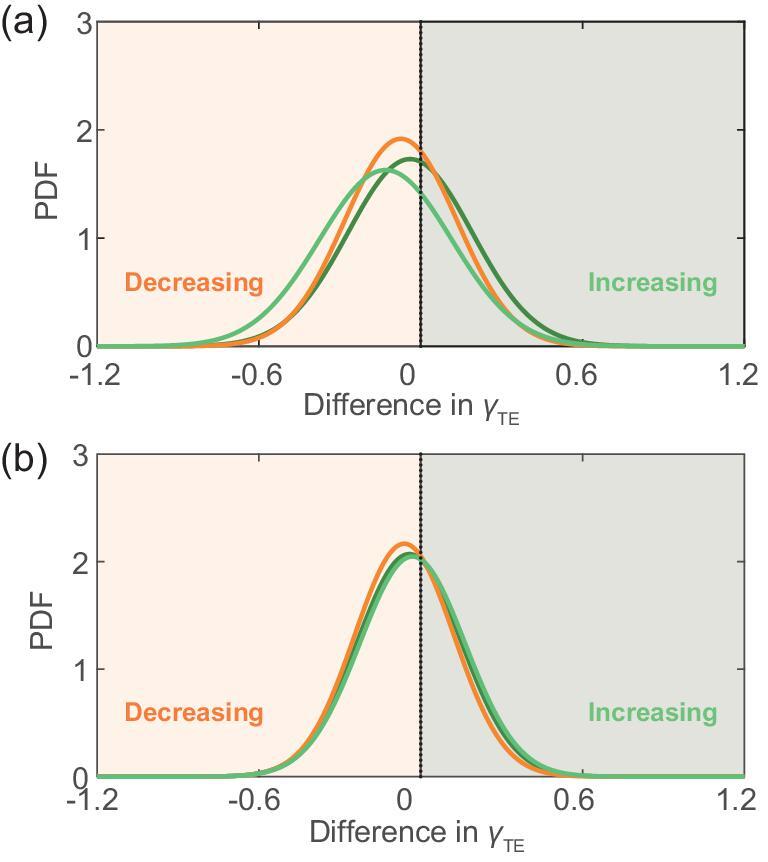
Probability density function (PDF) of the difference in the standard regression coefficients between mean growing season (April–October) Normalized Difference Vegetation Index (NDVI_GS_) and accumulated temperature exposures above (TE_H_) and below (TE_L_) the extremely high-temperature threshold. Extremely high temperature is defined as the 95th percentile of the daily temperature distribution for growing seasons during 1982–2012. The PDF shows the differences in the standard regression coefficients between NDVI_GS_ and growing season TE_H_ and TE_L_ for forest (dark green), shrubland (orange) and grassland (grass green) in the temperate (a) and the boreal (b) northern hemisphere. In this analysis, we only considered pixels with significant (*P* < 0.1) ridge regressions between NDVI_GS_, and total growing season precipitation, TE_H_ and TE_L_.

The divergent effects of the TE_H_ and TE_L_ on the NDVI_GS_ were most striking in temperate grasslands (Fig. 2). A more strongly negative standardized interannual response of the NDVI_GS_ to the TE_H_ than to the TE_L_ was found in ∼70% of temperate grasslands (Figs 1b, c and 2a). However, such divergent effects were less pronounced for temperate shrub and forests, with a greater decrease in the interannual response of the NDVI_GS_ to the TE_H_ than the TE_L_ found in only ∼53 and ∼58% of shrub lands and forests, respectively (Fig. 2a). Consistently divergent effects of the TE_H_ and TE_L_ on land-surface model simulations of mean growing season NPP (NPP_GS_) were also observed (Supplementary Fig. 7, available as Supplementary Data at *NSR* online). However, we discovered a more consistent decrease in the standardized interannual response of the NPP_GS_ to the TE_H_ than TE_L_ for all vegetation types in the temperate NH (Supplementary Fig. 8, available as Supplementary Data at *NSR* online).

Divergent effects of the TE_H_ and TE_L_ on the NDVI_GS_ were also observed in some parts of the boreal NH, particularly in northern Europe, northern America and northern Russia (Fig. 1b and c). The divergent effects of the TE_H_ and TE_L_ on the NDVI_GS_ were most prominent in shrub and forest in boreal NH (Fig. 2b). There was a much weaker positive response of vegetation growth to the TE_H_ than to the TE_L_ in these regions, indicating that the TE_H_ could restrict the positive response of vegetation growth to temperature, even in the boreal NH. This was verified by the consistent pattern of the divergent effects of the TE_H_ and TE_L_ on the NPP_GS_ in the boreal NH (Supplementary Fig. 8b, available as Supplementary Data at *NSR* online). It should be noted that these findings were not susceptible to arbitrarily different definitions of the growing season (i.e. April–October and May–September, respectively) for vegetation growth in the NH (Supplementary Figs 9 and 10, available as Supplementary Data at *NSR* online).

### Convex pattern in the response of vegetation growth to TE within different temperature ranges

A ridge regression was performed to explore the standardized interannual responses of the NDVI_GS_ to total growing season precipitation, mean growing season temperature and TE within different temperature ranges (at 1°C intervals) for different vegetation types in both the temperate and boreal NH during the period of 1982−2012. The ridge regression coefficients of TE within different temperature ranges were used to quantify the standardized interannual response of the NDVI_GS_ to differences in TE (${\gamma _{\rm TE}}$) over the period of 1982−2012. We observed generally positive and negative responses of the NDVI_GS_ to mean growing season temperature and total growing season precipitation, respectively, for forest (0.44 and –0.12, *P* < 0.05), shrub lands (0.34 and –0.09, *P* < 0.05) and grass [0.23 (*P* < 0.05) and –0.04 (*P* > 0.05)] in the boreal NH. In contrast, vegetation growth consistently displayed weak (*P* > 0.05) positive/negative and significantly (*P* < 0.05) positive responses to mean growing season temperature and total growing season precipitation, respectively, for forest (–0.04 and 0.23), shrub (0.05 and 0.23) and grass (0.09 and 0.32) in the temperate NH.

**Figure 3.**
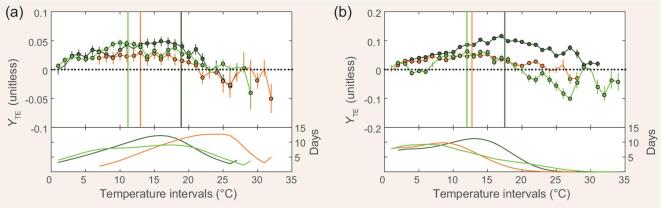
Non-linear relationship between the responses of the mean growing season (April–October) Normalized Difference Vegetation Index (NDVI_GS_) to temperature exposures (${\gamma _{\rm TE}}$) and TE within different temperature ranges. The non-linear relationship between the ${\gamma _{\rm TE}}$ of the NDVI_GS_ and TE within different temperature ranges (with 1°C intervals) for forest (dark green), shrubland (orange) and grassland (grass green) in the temperate (a) and the boreal (b) northern hemisphere for 1982–2012. Graphs at the top of each frame display the changes in the standardized response coefficients of NDVI_GS_ to a TE within a specific temperature range. The confidence intervals at the 95% level for ${\gamma _{\rm TE}}$ are marked by error bars. The curves are standardized so that the exposure-weighted impact is zero. Markers on lines indicate a significant response of NDVI_GS_ to TE. The vertical dashed lines at the top of each frame indicate temperature thresholds for the non-linear relationship between the ${\gamma _{\rm TE}}$ of NDVI_GS_ and TE for forest (dark green), shrubland (orange) and grassland (grass green). The average number of days within each 1°C interval for forest (dark green), shrubland (orange) and grassland (grass green) during growing seasons in the period of 1982–2012 is shown in the bottom part of each frame.

Given the magnitudes of the difference in hydrothermal conditions and vegetation structures among forest, shrubland and grassland in both the temperate and boreal NH, vegetation growth would not be expected to respond uniformly to TE among the different vegetation types. However, we identified a uniformly convex pattern in the ${\gamma _{\rm TE}}$ of the NDVI_GS_ in response to TE along with an increasing temperature gradient for all vegetation types in both the temperate and boreal NH (Fig. 3). This pattern showed a gradual increase in positive ${\gamma _{\rm TE}}$ until a temperature threshold, which was dependent on vegetation types, was reached. There was a weak positive ${\gamma _{\rm TE}}$ or even a negative ${\gamma _{\rm TE}}$ in response to a TE above the temperature thresholds. A consistent result was also found for NPP_GS_ (Supplementary Fig. 11, available as Supplementary Data at *NSR* online). Tree-ring analyses showed a consistent pattern in the relationship between the responses of the TRI and TE within different temperature ranges in the temperate NH, but no such convex pattern was observed in boreal TRI chronologies (Supplementary Fig. 12, available as Supplementary Data at *NSR* online).

Bent−Cable regression identified the different temperature thresholds in the relationships between the ${\gamma _{\rm TE}}$ of the NDVI_GS_ and TE within different temperature ranges for temperate (boreal) forest, shrubland and grassland, with those temperature thresholds centralized within the narrow ranges of ∼17–20 (15–19), ∼10–14 (10–15) and ∼10–12 (10–14)°C, respectively, for the two different growing season definitions (Supplementary Table 1, available as Supplementary Data at *NSR* online, Fig. 3 and Supplementary Fig. 13, available as Supplementary Data at *NSR* online). In the temperate NH, shrubland experienced much more exposure to warmer temperatures than grassland and forest, and there was a stronger negative ${\gamma _{\rm TE}}$ of the NDVI_GS_ and NPP_GS_ when the temperature exceeded the threshold (Fig. 3a and Supplementary Fig. 11, available as Supplementary Data at *NSR* online). In contrast, grassland in the boreal NH suffered from more exposure to warmer temperatures and there was a generally negative ${\gamma _{\rm TE}}$ of the NDVI_GS_ and NPP_GS_ for boreal grassland under exposure to temperatures in the right-hand tail of the daily temperature distribution curve, despite the large variations (Fig. 3b).

**Figure 4.**
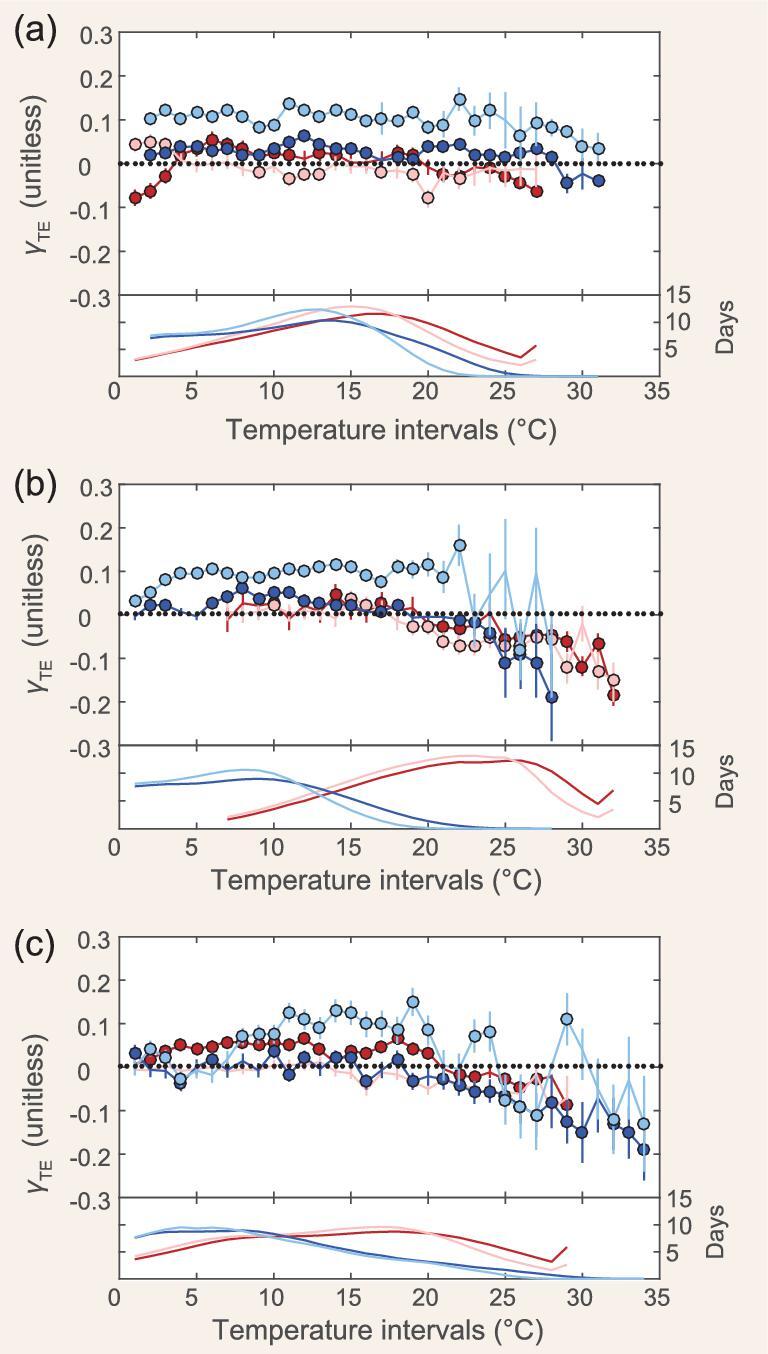
Comparison of the non-linear relationships between the responses of the mean growing season (April–October) Normalized Difference Vegetation Index (NDVI_GS_) to temperature exposures (${\gamma _{\rm TE}}$) and TE within different temperature ranges in years with more and fewer extremely high temperature (EHT) occurrences. The top part of each frame shows the non-linear relationships between the ${\gamma _{\rm TE}}$ of NDVI_GS_ and TE within different temperature ranges in years with more (darker lines) and fewer (lighter lines) EHT occurrences for forest (a), shrubland (b) and grassland (c) in the temperate (red lines) and the boreal (blue lines) northern hemisphere (NH) during 1982–2012. The confidence intervals at the 95% level for the ${\gamma _{\rm TE}}$ are marked by error bars. For each pixel within each vegetation type, we selected 7 years within the period of 1982–2012 with more EHT occurrences and 7 years with fewer EHT occurrences compared to the mean number of EHT occurrences over the same period. Lines at the bottom of each frame display the average number of days within different temperature ranges in cases with more than average (darker lines) and fewer than average (lighter lines) EHT occurrences in the temperate (red lines) and boreal (blue lines) NH for each vegetation type.

Finally, we compared the ${\gamma _{\rm TE}}$ of the NDVI_GS_ between years with more and fewer EHT events during 1982–2012 (see the ‘Methods’ section) for forest, shrubland and grassland, in both the temperate and boreal NH (Fig. 4). We observed a much larger divergence in the ${\gamma _{\rm TE}}$ of the NDVI_GS_ and NPP_GS_ in response to TE within the same temperature ranges between years with more and fewer EHT occurrences in the boreal NH than in the temperate NH for all vegetation types (Fig. 4 and Supplementary Fig. 14, available as Supplementary Data at *NSR* online). There was a much weaker positive ${\gamma _{\rm TE}}$ of the NDVI_GS_ and NPP_GS_ in years with more than average EHT occurrences than in years with fewer than average EHT occurrences for all vegetation types in the boreal NH (Fig. 4 and Supplementary Fig. 14, available as Supplementary Data at *NSR* online). However, such large divergence in the ${\gamma _{\rm TE}}$ of the NDVI_GS_ and NPP_GS_ were not observed in any vegetation types in the temperate NH. Instead, there was a generally consistent ${\gamma _{\rm TE}}$ of the NDVI_GS_ and NPP_GS_ in response to TE in years with more and fewer EHT occurrences (Fig. 4). However, there was a stronger negative ${\gamma _{\rm TE}}$ of the NDVI_GS_ in the temperate forest in response to TE in the right-hand tail of the temperature distribution in years with more than average EHT occurrences than in years with fewer than average EHT occurrences (Fig. 4a).

## DISCUSSION

Recent warming over the NH has been positively correlated with increasing terrestrial vegetation growth (also known as ‘northern greening’), which is mainly attributed to an increase in vegetation photosynthesis and an extension of the photosynthetic growing season, especially in the boreal NH [6,13–17]. However, we report here that exposures to temperatures beyond a vegetation type-dependent critical threshold consistently resulted in a weak positive ${\gamma _{\rm TE}}$ or even a negative ${\gamma _{\rm TE}}$ of vegetation growth for forest, shrubland and grassland in both the temperate and boreal NH, despite the differences in the trajectories of ${\gamma _{\rm TE}}$.

The mechanisms underlying the observed decrease in the ${\gamma _{\rm TE}}$ of vegetation growth in response to increasing temperature above these critical thresholds in both the temperate and the boreal NH could not be directly determined from the statistical analyses, and they remain unclear. Nevertheless, two possible mechanisms, namely warming-induced drought stress and a non-linear response of vegetation growth to increasing temperature, can partially explain the observed consistently convex pattern in the ${\gamma _{\rm TE}}$ of the NDVI_GS_/NPP_GS_ for all vegetation types in both the temperate and boreal NH. Temperature impacts on vegetation growth directly and indirectly through its effects on plant physiological processes and hydrothermal conditions. In water-limited temperate regions, warmer temperature and an increase in the number and/or duration of EHT events can intensify drought stress (Supplementary Figs 15 and 16, available as Supplementary Data at *NSR* online) and thus reduce the availability of water to support vegetation growth during the warm season [18] (Supplementary Fig. 16, available as Supplementary Data at *NSR* online). This mechanism also makes a major contribution to the widely documented decline in vegetation growth and the observed weakening positive relationship between vegetation growth and temperature variations in such regions [5,19–21]. EHT and/or EHT-induced megadroughts can even trigger widespread lagged growth recovery and even forest die-off in water-limited regions [18,22], which is mainly attributed to hydraulic failure and/or carbon starvation [20,21,23,24]. Our tree-ring analyses consistently indicated that a TE beyond a certain threshold (Supplementary Fig. 12, available as Supplementary Data at *NSR* online) resulted in a weak positive or even a negative ${\gamma _{\rm TE}}$ for tree growth in the temperate NH, even though most of the TRI chronologies used in this study were primarily produced for reconstructions of past temperature, and the trees sampled were generally species limited by warm-season temperatures (e.g. [25]).

Growth of all three vegetation types in the boreal NH was more susceptible to TE under high temperatures than in the temperate NH. Our findings were consistent with those of previous studies showing that the physiological processes of plants grown in water-limited regions (e.g. temperate shrublands and grasslands) have low drought sensitivity, but are more flexible in adapting to high temperatures and/or drought stress [26,27]. Although previous studies have shown that drought generally has a major influence on vegetation growth in water-limited regions, this does not necessarily mean that plant communities in these regions are more vulnerable to warming-mediated drought limitation than those in humid regions [27,28]. An increase in the occurrence of EHT events in boreal regions can also result in drier conditions or even negative water balances (e.g. in grasslands in the boreal NH) (Supplementary Figs 15b and 16, available as Supplementary Data at *NSR* online).

Water availability and temperature can interact to regulate the response of vegetation growth to TEs across diverse bioclimatic regions [29,30]. For example, there was a strong negative ${\gamma _{\rm TE}}$ of the NDVI_GS_ in response to TE within the right-hand tail of the temperature distribution for boreal grass in years with more EHT occurrences compared to years with fewer EHT occurrences (Fig. 4c). The vegetation growth in the boreal region was also vulnerable to drought stress [27,30] and recent long-lasting global-change-type drought stress in parts of the boreal regions has triggered local forest die-off [20,31]. Vegetation in humid regions in the boreal NH is particularly sensitive to drought and responds to it over short time scales [27,28]. Furthermore, drought can significantly mediate the temperature sensitivity of vegetation growth, and increasing evidence illustrated that drought stress may reduce, or even reverse, the potential benefits of climate warming on vegetation growth in boreal regions [30], but enhance the temperature sensitivity in tropical regions [29]. Evidence from tree-ring analyses has also confirmed that drought in parts of the boreal regions has resulted in a significantly weaker positive relationship between tree growth and temperature [32]. However, we did not observe a weakening positive ${\gamma _{\rm TE}}$ of the TRI in the boreal NH in response to TE over an increasing temperature gradient, when all the chronologies in the boreal NH for the period 1982–2012 were considered (Supplementary Fig. 12, available as Supplementary Data at *NSR* online). This may be attributable to the considerable variations in local climate conditions and forest histories (e.g. management) among sampling sites. In addition, trees in the northern taiga have been shown to allocate more carbon to their stems and roots, and less carbon to leaves, under the warmer and drier conditions of recent years [33], which could also partly explain the different trajectories in the ${\gamma _{\rm TE}}$ between the NDVI_GS_ and TRI in boreal forest (Fig. 3b and Supplementary Fig. 12, available as Supplementary Data at *NSR* online).

Another mechanism underlying the observed decrease in the ${\gamma _{\rm TE}}$ of vegetation growth in response to increasing temperatures is the non-linear response of photosynthesis to increasing temperature, with the downregulation of photosynthesis occurring once the optimal temperature threshold was crossed. This mimicked the documented convex curve of the response of photosynthesis to increasing temperature [34–36]. Such non-linear temperature responses of photosynthesis have been reported to be responsible for the observed weakening positive relationship between vegetation growth and mean growing season temperature variations in the NH [5]. Importantly, the non-linear temperature response of vegetation photosynthesis can be largely mediated by warmer temperature induced intensified drought stress [37].

We suggest that plant trait-mediated different ecophysiological properties among different vegetation types also contribute to the different trajectories in the ${\gamma _{\rm TE}}$ of the NDVI_GS_/NPP_GS_. Our meta-analysis revealed that deep-rooted forests in the temperate NH generally adopt a conservative water-use strategy and have a high capacity for acquiring deep soil water (Supplementary Table 2 and Supplementary Fig. 17, available as Supplementary Data at *NSR* online). Shrubs in water-limited regions generally have an adaptive water-use strategy and can acquire soil water from different soil layers during the growing season (Supplementary Fig. 17, available as Supplementary Data at *NSR* online). The growth of these two vegetation types is thus regarded as being more resistant to drought and/or EHT events than shallow-rooted grass, particularly in water-limited regions. However, an adaptive water-use strategy cannot fully explain the observed strongly negative ${\gamma _{\rm TE}}$ of NDVI_GS_/NPP_GS_ for temperate shrubland in response to the exposure to temperatures in the right-hand tail of the distribution in years with either more or fewer EHT occurrences (Figs 3a and 4b). This is most likely because the most severe water deficit was observed in temperate shrubland regions (Supplementary Fig. 16, available as Supplementary Data at *NSR* online). Shallow-rooted grass ecosystems generally use water from the top soil layers (Supplementary Fig. 17 and Supplementary Table 2, available as Supplementary Data at *NSR* online) and tend to be more vulnerable to EHT events [38,39]. This partly explains the observed lower and higher temperature thresholds in the non-linear ${\gamma _{\rm TE}}$ of NDVI_GS_/NPP_GS_ for grass and forest, respectively, in both the temperate and the boreal regions.

The physiological performance of NH vegetation in warmer and more extreme climates can also be affected by non-climate factors. For example, the rising CO_2_ concentration and availability of background nutrition could act together to mediate the responses of vegetation growth to drought stress and EHT events by regulating stomatal conductance and nutrient tolerance [40–42]. Previous studies have shown that an increasing CO_2_ concentration can increase whole-plant water-use efficiency, and thus partly alleviate the harmful effects of extreme drought or heat waves on vegetation productivity, especially in water-limited regions [43]. However, such benefits are susceptible to the seasonality of water availability and its effect on vegetation functioning [40,44,45], as well as the changing community composition (e.g. species composition) [42].

An improved understanding of the mechanisms underlying the impacts of warmer temperature distributions on vegetation growth is crucial when attempting to predict future ecosystem functioning, the global carbon cycle and subsequent climate feedbacks. Further research into the role of warmer temperature distributions on different terrestrial ecosystems is urgently needed, particularly studies based on long-term ecological experiments and diagnostic simulations using improved land-surface models.

## METHODS

### Vegetation growth, climate and stable isotope datasets

The latest (third) version of the biweekly NDVI data, with a spatial resolution of 0.083°, produced from the Advanced Very High Resolution Radiometer (AVHRR) observations during 1982–2012 (i.e. GIMMS NDVI_3g_), was obtained from the Global Inventory Modeling and Mapping Studies (GIMMS) group (https://nex.nasa.gov/nex/projects/1349/). We resampled the NDVI data into a spatial resolution of 0.5° to match the climate data used. The GIMMS NDVI_3g_ dataset has been processed to account for orbital drift, sensor degradation, inter-sensor differences, cloud cover, zenith angle and volcanic aerosols [46]. This dataset has been widely used to monitor interannual variations in NH terrestrial vegetation growth and productivity [5].

Gridded monthly NPP from 1982 to 2010 were obtained from the TRENDY project (http://dgvm.ceh.ac.uk/node/9). We obtained the NPP outputs for the S2 scenario (in which both the climate and CO_2_ concentration are changed) from four land-surface models: LPJ, LPJ-GUESS, ORCHIDEE and VEGAS. These four land-surface models provided monthly NPP simulations at a spatial resolution of 0.5°.

A total of 446 standard TRI chronologies were used in this study to investigate the relationships between interannual tree-growth activity and TE in the temperate (30°–50° N) and boreal (50°–70° N) NH (Supplementary Table 3, available as Supplementary Data at *NSR* online). Of these TRI series, 433 were selected from the International Tree-Ring Data Bank (ITRDB, http://www.ncdc.noaa.gov/data-access/paleoclimatology-data/datasets/tree-ring) (Supplementary Text ST1, available as Supplementary Data at *NSR* online). We provided another 13 standard TRI chronologies. These standard TRI chronologies were built following standard dendrochronology procedures [47].

The global gridded sub-daily climate data (including temperature, precipitation and short-wave solar radiation) between 1982 and 2012, with a spatial resolution of 0.5° and a time resolution of 6 hours, was obtained from the Climatic Research Unit—National Centers for Environmental Prediction (CRUNCEP) dataset (version 5, http://dods.extra.cea.fr/data/p529viov/cruncep/readme.htm). Global Standard Precipitation-Evapotranspiration Index (SPEI) data, with a time scale of 6 months over the period of 1982–2012, was compiled from SPEIbase v2.3 (http://sac.csic.es/spei/database.html) [48]. The monthly gridded precipitation and potential evapotranspiration data, with a spatial resolution of 0.5°, were also obtained from the CRU TS3.22 dataset (http://www.cru.uea.ac.uk/cru/data/hrg/) to calculate the mean growing season water deficits (*WD*) between 1982 and 2012, using Equation (1):

(1) \begin{equation*}W{D_i} = GS{P_i} - PE{T_i},\end{equation*}

where $GS{P_i}$ and $PE{T_i}$ were the total precipitation and potential evapotranspiration during the growing season for the *i_th_* year, respectively.

Isotope-derived plant water-uptake fractions from different soil layers throughout the growing season for trees, shrubs and grasses in the temperate NH were obtained from literature surveys. Three different soil layers were roughly defined for the purposes of this study, with the shallow, middle and deep soil layers corresponding to 0−20/30, 20/30−50/70 and >50/70 cm, respectively. In some studies, seasonal water-uptake fractions were investigated. In such cases, we simply calculated the mean plant water-uptake fractions during the growing season from the three different soil layers by averaging the seasonal water-uptake fractions, although it should be noted that there is a large seasonal variation in plant water uptake. The details of the survey undertaken in the study are listed in Supplementary Table 2, available as Supplementary Data at *NSR* online.

## Statistical analyses

### Relationships between mean growing season vegetation growth and climate factors

We defined the 95th percentile of the daily temperature distribution of growing seasons during the period of 1982–2012 as the EHT threshold in each grid. We then calculated the accumulated TE above and below the EHT threshold (TE_H_ and TE_L_, respectively) in each growing season for the period of 1982–2012 for each grid. Ridge regression was performed to identify the interannual relationships between NDVI_GS_/NPP_GS_ and different sets of climate variables. The first model only includes the total growing season precipitation, mean growing season temperature and mean growing season solar radiation. An alternative ridge regression model (i.e. Model 2) between NDVI_GS_/NPP_GS_ and total growing season precipitation, mean growing season temperature, mean growing season solar radiation, and the TE_H_ and TE_L_ was also evaluated. The AIC was introduced to evaluate the performance of the two alternative models [49]. We found that the inclusion of mean growing season solar radiation and mean growing season temperature did not significantly improve the second model performance (Supplementary Figs 2 and 3, available as Supplementary Data at *NSR* online). Therefore, the mean growing season solar radiation and mean growing season temperature were not included in our final analyses. Further, we compared the ridge regression results with TE_H_ and TE_L_ calculated using different definitions of EHT, as well as results from simple multivariate linear regression (Supplementary Text ST2, available as Supplementary Data at *NSR* online). All variables were normalized prior to conducting ridge regression and multivariate linear regression analyses. Regions with multi-year mean NDVI values <0.1 during 1982−2012 were discarded from our analyses.

### Non-linear responses of vegetation growth to TE

The non-linear responses of vegetation growth to climate factors during the period of 1982–2012 were investigated for different vegetation types using Equation (2):

(2) \begin{equation*} {y_{it}} = \smallint\nolimits_{\underline h} ^{\overline{h}} {\gamma _h}{\emptyset _{it}}\left( h \right)\,dh + \delta {P_{it}} + \theta {T_{it}} + \epsilon , \end{equation*}

where ${y_{it}}$ was the vegetation growth proxy represented by NDVI_GS_, NPP_GS_ or TRI in region *i* and year *t* (if applicable). ${\gamma _h}$ and ${\emptyset _{it}}( h )$ were the response coefficients of the vegetation growth proxy to TE and the time distribution of TE over the growing season in region *i* and year *t*, respectively. The temperature data covered the period of 1982–2012 for region *i* between the lower boundary $\underline{h} $ and upper boundary $\bar{h}$. A quadratic function representing total growing season precipitation and linear function representing mean growing season temperature and random error are denoted as *P_it_*, *T_it_* and $\epsilon $, respectively, whereas *δ* and *θ* are the corresponding coefficients for the total growing season precipitation and mean growing season temperature (Supplementary Text ST3, available as Supplementary Data at *NSR* online). We fixed the growing season to be April–October for vegetation in both the temperate and boreal NH. Another growing season definition, May–September, was also analysed to verify the robustness of our conclusions. However, the effects of temporal changes in CO_2_ concentrations on the responses of vegetation growth to TE within different temperature ranges were not considered in our analyses (Supplementary Text ST4, available as Supplementary Data at *NSR* online).

Specifically, we approximated the time distribution of TE in Equation (2) with a 1°C interval, as shown in Equation (3):

(3) \begin{eqnarray*} {y_{it}} &=& \sum \nolimits_{j = \underline{h} }^{\bar{h}} {\gamma _j} \left[ {{\emptyset _{it}}\left( {h + 1} \right) - {\emptyset _{it}}\left( h \right)} \right]\nonumber\\ && +\, \delta {P_{it}} + \theta {T_{it}} + \epsilon , \end{eqnarray*}

where ${\gamma _j}$ was the regression coefficient of the vegetation growth proxy to TE within the *j_th_* temperature bin estimated by the ridge regression. The lower boundary $\underline{h} $ and upper boundary $\bar{h}$ for TE during the growing season were calculated for forests, shrubland and grassland, respectively (Supplementary Text ST5, available as Supplementary Data at *NSR* online). Bent–Cable regression analyses were performed to identify possible temperature thresholds in the non-linear relationship between TE and the corresponding response coefficients of the vegetation growth proxy to TE [50].

We compared the non-linear relationships between the responses of NDVI_GS_ to TE (${\gamma _{\rm TE}}$) in years with more and fewer EHT occurrences for forests, shrubland and grassland in both the temperate and boreal NH. We selected 7 years with more EHT occurrences and 7 years with fewer EHT occurrences relative to the mean number of EHT occurrences during 1982−2012 in each grid (Supplementary Text ST6, available as Supplementary Data at *NSR* online).

## Supplementary Material

nwy158_Supplemental_File
