# Supplementary material for: Exposures to temperature beyond threshold disproportionately reduce vegetation growth in the northern hemisphere
Source: Natl Sci Rev. 2018 Dec 22;6(4):786–95. doi: 10.1093/nsr/nwy158 (PMC8291599; doi:10.1093/nsr/nwy158)
Supplement: nwy158_Supplemental_File [file nwy158_supplemental_file.docx]

To be submitted to *National Science Review*

Supplementary materials for

# Exposures to temperature beyond threshold disproportionately reduce vegetation growth in the northern hemisphere

Xiuchen Wu^a,b,1^ , Weichao Guo^c^, Hongyan Liu^c,1^, Xiaoyan Li^a,b^, Changhui Peng^d^, Craig D. Allen^e^, Cicheng Zhang^b^, Pei Wang^a,b^, Tingting Pei^b^, Yujun Ma^a,b^, Yuhong Tian^b^, Zhaoliang Song^f^, Wenquan Zhu^b^, Yang Wang^b^, Zongshan Li^g^, Deliang Chen^h^

1. State Key Laboratory of Earth Surface Processes and Resource Ecology, Beijing Normal University, Beijing, 100875, China
2. College of Resources Science and Technology, Beijing Normal University, Beijing, 100875, China
3. College of Urban and Environmental Science, Peking University, Beijing, 100871, China
4. Institute of Environment Sciences, University of Quebec at Montreal, Quebec, Canada
5. U.S. Geological Survey, Fort Collins Science Center, Jemez Mountains Field Station, Los Alamos, New Mexico, 87544, USA
6. Institute of the Surface-Earth System Science Research, Tianjin University, Tianjin, 300072, China
7. State Key Laboratory of Urban and Regional Ecology, Research Center for Eco-Environmental Sciences, Chinese Academy of Sciences, Beijing, 100085, China
8. Regional Climate Group, Department of Earth Sciences, University of Gothenburg, Gothenburg, 460, Sweden.

1. Corresponding author: Xiuchen Wu, Faculty of Geophysical Science, Beijing Normal University, Beijing, 100875, China. Tel: 0086-10-58807052, [xiuchen.wu@bnu.edu.cn](mailto:xiuchen.wu@bnu.edu.cn); Hongyan Liu, College of Urban and Environmental Science, Peking University, Beijing, 100871, China. Tel: 0086-10-62759319, [lhy@urban.pku.edu.cn](mailto:lhy@urban.pku.edu.cn).

This file contains:

1. Supplementary Text
2. Supplementary Table 1-3.
3. Supplementary Figure 1-17.
4. References

Supplementary text

**Vegetation growth, climate, and stable isotope datasets**

**ST1:** We selected 433 TRI chronologies based on three criteria: 1) they contained detailed records of latitude, longitude, elevation, species name, and sample depth; 2) the chronologies covered at least the period of 1982–1995 or were extended to include more recent years (i.e., the early 21^st^ century); and 3) the number of samples for each site-year was greater than five.

**Statistical analyses**

**Relationships between mean growing season vegetation growth and climate factors.**

**ST2:** We compared the ridge regression results with TE_H_ and TE_L_ calculated using different definitions of EHT, i.e., the 90^th^ and 99^th^ percentile of daily temperature distribution of growing seasons in period of 1982-2012, to validate our conclusions. We presented the results with EHT definition of 95^th^ percentile in the main text, and showed other results in the supplementary materials. We further compared the spatial pattern in the responses of mean growing-season NDVI to total growing season precipitation, and accumulative TE_H_ and TE_L_ during growing-season from both ridge regression and simple multivariate linear regression.

**Nonlinear responses of vegetation growth to TE**

**ST3**: The inclusion of mean growing season temperature in this analysis can eliminate its interactive effects on the responses of mean growing-season vegetation growth to TE within different temperature ranges.

**ST4:** We aimed to quantify the general patterns in the responses of vegetation growth to TE within different temperature ranges, among different vegetation types in both the temperate and boreal NH, taking the period of 1982–2012 as the analysis time domain. We focused less on the temporal changes in the response of vegetation growth to the variations of climate, and therefore temporal changes in CO_2_ concentration could impact on the temporal pattern in the responses of vegetation growth to climate variations.

**ST5**: Three major vegetation types are reclassified, i.e., forests (evergreen needle leaf and broadleaf forest, deciduous needle leaf and broadleaf forest, and mixed forest), shrubland (closed and open shrublands, woody savanna, and savanna), and grassland, in the temperate and the boreal NH based on the Moderate Resolution Imaging Spectroradiometer land cover product MOD12C1 (<http://glcf.umd.edu/data/lc/>), which identifies 17 land cover classes defined by the International Geosphere-Biosphere Program (IGBP) scheme.

**ST6:** Then, we compiled the NDVI_GS_, and TE within different temperature ranges, mean growing season temperature, and total growing season precipitation for years with more than average EHT occurrences and years with fewer than average EHT occurrences, respectively, for each region of forest, shrub, and grass. All variables in each pixel during 1982−2012 were first normalized prior to analysis for each vegetation type in either temperate NH or boreal NH.

Supplementary Table 1. Temperature thresholds (°C) in the relationships between the interannual response of mean growing season Normalized Difference Vegetation Index (NDVI_GS_) to temperature exposures (TE), and TE within different temperature intervals, for different biomes in the temperate and the boreal Northern Hemisphere (NH).

| Growing-Season^¶^ | Climate Region^†^ | Biome^§^ | | | | | | | |
| --- | --- | --- | --- | --- | --- | --- | --- | --- | --- |
|  |  | Forest | |  | Shrubland | |  | Grassland | |
| April-October | Temperate NH | 18.90^ǂ^ | 2.1^ǁ^ |  | 12.85 | 1.9 |  | 11.45 | 1.3 |
|  | Boreal NH | 17.5 | 1.9 |  | 12.65 | 1.7 |  | 12.20 | 1.5 |
| May-September | Temperate NH | 18.5 | 2.4 |  | NaN | NaN |  | 10.8 | 1.7 |
|  | Boreal NH | 17.2 | 2.7 |  | 12.9 | 2.4 |  | 12.0 | 2.5 |

^¶^ In this study, two different definitions of growing season were analyzed in order to verify whether our conclusions are affected by arbitrary definitions of growing seasons.

^†^ Biomes in the temperate (30º-50º N) and the boreal (50º-70º N) Northern Hemisphere (NH) were analyzed separately.

^§^ Bent−Cable regressions were performed to identify the temperature thresholds (marked by ǂ) and their confidence intervals (marked by ǁ) for the relationships between the interannual sensitivity of NDVI_GS_ to TE, and TE within different temperature intervals, in forest, shrub land and grassland. NaN indicates that no obvious temperature thresholds could be determined.

Supplementary Table 2. Details of the sites surveyed for vegetation water uptake.

| Biome | Type of Vegetation | Species | Water uptake | | | Latitude | Longitude | Elevation (m) | MAT (^o^C) | MAP (mm) | PET (mm) | Ecosystem | Country | References |
| --- | --- | --- | --- | --- | --- | --- | --- | --- | --- | --- | --- | --- | --- | --- |
|  |  |  | Shallow layer^*^ (%) | Middle layer^†^ (%) | Deep layer^‡^ (%) |  |  |  |  |  |  |  |  |  |
| Forest | Temperate woodland tree | *Quercus macrocarpa* | 20 | 0 | 80 | 41.55°N | 93.28°W | \ | \ | 840 | \ | woodland/shrubland | USA | Asbjornsen et al. (2007) |
| Forest | Temperate woodland tree | *Ulmus americana* | 20 | 0 | 80 | 41.55°N | 93.28°W | \ | \ | 840 | \ | woodland/shrubland | USA | Asbjornsen et al. (2007) |
| Forest | Deciduous tree | *Salix alba* | 22.6 | 40.175 | 37.3 | 46.23°N | 7.36°E | 510 | \ | 587 | \ | temperate deciduous tree | Switzerland | Bertrand et al. (2014) |
| Forest | Deciduous tree | *Prunus avium* | 11.2 | 30.9 | 62.9 | 46.23°N | 7.36°E | 510 | \ | 587 | \ | temperate deciduous tree | Switzerland | Bertrand et al. (2014) |
| Forest | Deciduous tree | *Populusnigra* | 38 | 30.775 | 31.4 | 46.23°N | 7.36°E | 510 | \ | 587 | \ | temperate deciduous tree | Switzerland | Bertrand et al. (2014) |
| Forest | Deciduous tree | *Alnus glutinosa* | 21.2 | 42.875 | 35.3 | 46.23°N | 7.36°E | 510 | \ | 587 | \ | temperate deciduous tree | Switzerland | Bertrand et al. (2014) |
| Forest | Coniferous | *Pinus Sylvestris* | 22.9 | 70.575 | 24.1 | 46.23°N | 7.36°E | 510 | \ | 587 | \ | temperate deciduous tree | Switzerland | Bertrand et al. (2014) |
| Forest | Deciduous tree | *Populus euphratica* | 1.2 | 6.8 | 92 | 42.68°N | 102.13°E | \ | 8.6 | 38.49 | 3467 | Riparian forest | China | Liu et al. (2015) |
| Forest | Evergreen tree | *Pinus tabuliformis* | 3.5 | 10.5 | 86 | 39.12°N | 109.90°E | \ | \ | 450 | 1800 | temperature forest/desert plant | China | Li et al. (2013) |
| Forest | Deciduous tree | *Populus simonii* | 3.5 | 4.5 | 92 | 39.12°N | 109.90°E | \ | \ | 450 | 1800 | temperature forest/desert plant | China | Li et al. (2013) |
| Shrubland | Deciduous, perennial subshrub | *Artemisia oxycephala* | 68.8±23.9 | 18.2±14.7 | 13.0±10.7 | 36.78°N | 100.78°E | 3224 | 0.7 | 368 | 1484 | alpine meadow | China | Wu et al. (2016) |
| Shrubland | Deciduous, perennial shrub | *Hippophae rhamnoides* | 31.5±30.6 | 34.8±32.5 | 33.6±32.5 | 36.78°N | 100.78°E | 3224 | 0.7 | 368 | 1484 | alpine meadow | China | Wu et al. (2016) |
| Shrubland | Deciduous, perennial subshrub | *Reaumuria songarica* | 38.2±16.2 | 37.1±17.2 | 24.6±18.1 | 39.32°N | 100.08°E | 1700 | \ | 117 | \ | Desert | China | our group (unpublished data) |
| Shrubland | Deciduous, perennial subshrub | *Nitraria sphaerocarpa* | 22.5±15.0 | 25.6±14.7 | 52.3±26.0 | 39.33°N | 100.09°E | 1700 | \ | 117 | \ | Desert | China | our group (unpublished data) |
| Shrubland | Deciduous, perennial shrub | *Artemisia tridentata* | 10 | 32 | 58 | 40.28°N | 112.47°W | 1660 | \ | 264 | \ | Savanna | USA | Prieto et al. (2014) |
| Shrubland | Deciduous, perennial shrub | *Haloxylon ammodendron* | 0 | 3 | 97 | 44.37°N | 87.92°E | 435 | \ | 130 | 2000 | derset | China | Dai et al. (2015) |
| Shrubland | Deciduous, perennial shrub | *Haloxylon persicum* | 5 | 13 | 82 | 44.37°N | 87.92°E | 435 | \ | 130 | 2000 | derset | China | Dai et al. (2015) |
| Shrubland | Deciduous, perennial shrub | *Haloxylon ammodendron* | 0 | 5 | 95 | 44.37°N | 87.92° E | 435 | 6.6 | 150 | 2000 | temperate desert | China | Dai et al. (2014) |
| Shrubland | Deciduous, perennial shrub | *Caragana microphylla* | 34.7±7.8 | 32.4±7.4 | 32.9±4.14 | 42.17°N | 115.82°E | \ | 1.6 | 407 | 1900 | temperate meadow | China | Zheng et al. (2015) |
| Shrubland | Deciduous, perennial shrub | *Ceanothus spp.* | 12.2 | 87.8 | 0 | 39.08°N | 96.51°W | \ | \ | \ | \ | temperate grassland | USA | Nippert et al. (2007) |
| Shrubland | Deciduous, perennial shrub | *Amorpha canescens* | 17.7 | 82.3 | 0 | 39.08°N | 96.51°W | \ | \ | \ | \ | temperate grassland | USA | Nippert et al. (2007) |
| Shrubland | Deciduous, perennial shrub | *Caragana intermedia* | 44 | 40 | 16 | 36.27°N | 100.27°E | 2871 | 2.4 | 246.3 | 1716 | Alipine shrubland | China | Jia et al. (2012) |
| Shrubland | Deciduous, perennial shrub | *Salix psammophila* | 10 | 24.5 | 65.5 | 39.12°N | 109.90°E | \ | \ | 450 | 1800 | temperature forest/desert plant | China | Li et al. (2013) |
| Shrubland | Deciduous, perennial shrub | *Artemisia desertorum* | 3.5 | 21 | 75.5 | 39.12°N | 109.90°E | \ | \ | 450 | 1800 | temperature forest/desert plant | China | Li et al. (2013) |
| Shrubland | Deciduous, perennial shrub | *Caragana korshinskii* | 11.5 | 35.5 | 53 | 39.12°N | 109.90°E | \ | \ | 450 | 1800 | temperature forest/desert plant | China | Li et al. (2013) |
| Grassland | Perennial herb | *Carex moorcroftii* | 59.5±23.8 | 26.4±22.9 | 13.9±6.2 | 36.78°N | 100.78°E | 3224 | 0.7 | 368 | 1484 | Alpine meadow | China | Wu et al. (2016) |
| Grassland | Perennial herb | *Astragalus adsurgens* | 33.6±31.8 | 26.4±22.9 | 13.9±6.2 | 36.78°N | 100.78°E | 3224 | 0.7 | 368 | 1484 | Alpine meadow | China | Wu et al. (2016) |
| Grassland | Perennial herb | *Andropogon gerardii* | 36 | 64 | 0 | 41.55°N | 93.28°W | \ | \ | 840 | \ | woodland/shrubland | USA | Asbjornsen et al. (2007) |
| Grassland | Herb | *Phleum pratense* | 90 | 6 | 4 | 47.21°N | 8.41°E | 393 | \ | \ | \ | sub-alpine grassland | Switzerland | Prechsl et al. (2015) |
| Grassland | Herb | *Trisetum flavescens* | 65 | 23 | 12 | 46.58°N | 9.79°E | 1978 | \ | \ | \ | managed grassland | Switzerland | Prechsl et al. (2015) |
| Grassland | Herb | *Andropogon gerardii* | 14.3 | 85.7 | 0 | 39.08°N | 96.51°W | \ | \ | \ | \ | temperate grassland | USA | Nippert et al. (2007) |
| Grassland | Herb | *Schizachyrium scoparium* | 10.7 | 89.3 | 0 | 39.08°N | 96.51°W | \ | \ | \ | \ | temperate grassland | USA | Nippert et al. (2007) |
| Grassland | Herb | *Sorghastrum nutans* | 12.2 | 87.8 | 0 | 39.08°N | 96.51°W | \ | \ | \ | \ | temperate grassland | USA | Nippert et al. (2007) |
| Grassland | Herb | *Lespedeza capitata* | 15.5 | 85.5 | 0 | 39.08°N | 96.51°W | \ | \ | \ | \ | temperate grassland | USA | Nippert et al. (2007) |
| Grassland | Herb | *Vernonia baldwinii* | 13.3 | 86.7 | 0 | 39.08°N | 96.51°W | \ | \ | \ | \ | temperate grassland | USA | Nippert et al. (2007) |

Note: Water uptake fraction data are shown here as mean ± standard deviation (if available). \ means that there are no records in the relevant literature. The definitions of shallow, middle and deep layers are different among different studies. In our study we roughly defined the shallow, middle and deep soil layers as follows:

^*^ Shallow soil layer 0-20/30 cm; ^†^ middle soil layer 20/30-50/70 cm and ^‡^ deep soil layer > 50/70 cm.

Supplementary Table 3. Background information for the standard tree ring chronologies used in this study.

| SiteID^*^ | Latitude | Longitude | Elevation | Start Year | End Year | Species^ǂ^ | Data Source^†^ |
| --- | --- | --- | --- | --- | --- | --- | --- |
| japa008 | 43.77 | 142.55 | 1350 | 1532 | 1997 | PCGN | ITRDB |
| japa011 | 44.02 | 143.83 | 200 | 1693 | 2003 | PCGN | ITRDB |
| kore001 | 38.13 | 128.47 | 1500 | 1657 | 1998 | PIKO | ITRDB |
| leba001 | 33.68 | 35.68 | 1775 | 1829 | 2002 | CDLI | ITRDB |
| leba002 | 34.47 | 36.23 | 1175 | 1722 | 2001 | ABCI | ITRDB |
| leba003 | 34.30 | 35.98 | 1640 | 1809 | 2001 | CDLI | ITRDB |
| leba004 | 34.23 | 36.03 | 1900 | 1382 | 2002 | CDLI | ITRDB |
| leba005 | 34.13 | 35.82 | 1780 | 1778 | 2002 | CDLI | ITRDB |
| leba006 | 33.67 | 35.68 | 1720 | 1730 | 2002 | CDLI | ITRDB |
| mong003 | 48.30 | 98.93 | 2420 | 900 | 1999 | PISI | ITRDB |
| mong005 | 48.57 | 110.55 | 1070 | 1651 | 1996 | PISY | ITRDB |
| mong006 | 47.78 | 107.50 | 1415 | 1582 | 1996 | LASI | ITRDB |
| mong008 | 49.37 | 94.88 | 2229 | 1641 | 1998 | PISI | ITRDB |
| mong009 | 49.92 | 91.57 | 2500 | 1326 | 1998 | LASI | ITRDB |
| mong010 | 47.27 | 100.03 | 2500 | 1363 | 1999 | LASI | ITRDB |
| mong011 | 48.15 | 100.28 | 1900 | 1513 | 2001 | LASI | ITRDB |
| mong012 | 48.98 | 103.23 | 1400 | 1511 | 2002 | LASI | ITRDB |
| mong013 | 48.77 | 97.12 | 1841 | 1638 | 1998 | LASI | ITRDB |
| mong014 | 49.48 | 100.83 | 1800 | 1557 | 2002 | LASI | ITRDB |
| mong015 | 48.17 | 99.87 | 2060 | 1340 | 2000 | LASI | ITRDB |
| russ019 | 72.50 | 105.17 | 40 | 1580 | 1997 | LAGM | ITRDB |
| russ192 | 68.42 | 35.28 | 220 | 1577 | 1997 | PISY | ITRDB |
| russ215 | 54.20 | 90.83 | / | 1767 | 1996 | LAGM | ITRDB |
| russ219 | 43.25 | 145.98 | / | 1585 | 2000 | QUMO | ITRDB |
| russ240 | 52.48 | 98.97 | 2170 | 1603 | 2013 | LASI | ITRDB |
| russ241 | 52.40 | 98.68 | 2020 | 1523 | 2013 | LASI | ITRDB |
| syri001 | 35.60 | 36.22 | 1450 | 1837 | 2001 | CDLI | ITRDB |
| syri002 | 35.57 | 36.20 | 1450 | 1795 | 2001 | ABCI | ITRDB |
| syri003 | 35.78 | 36.02 | 480 | 1882 | 2001 | PIBR | ITRDB |
| cana150 | 49.87 | -118.85 | 1700 | 1689 | 1998 | PCEN | ITRDB |
| cana151 | 49.87 | -118.85 | 1700 | 1669 | 1998 | PICO | ITRDB |
| cana152 | 49.87 | -118.85 | 1700 | 1725 | 1998 | ABLA | ITRDB |
| cana161 | 51.03 | -119.05 | 1900 | 1710 | 1996 | PCEN | ITRDB |
| cana162 | 51.03 | -119.05 | 1900 | 1773 | 1996 | ABLA | ITRDB |
| cana174 | 50.22 | -126.35 | 1005 | 1394 | 1999 | ABAM | ITRDB |
| cana175 | 50.22 | -126.35 | 1005 | 1200 | 1999 | CHNO | ITRDB |
| cana195 | 49.83 | -97.20 | 230 | 1286 | 1999 | QUMA | ITRDB |
| cana211 | 61.90 | -140.72 | 731 | 1679 | 1999 | PCGL | ITRDB |
| cana221 | 53.95 | -106.33 | 611 | 1682 | 1997 | LALA | ITRDB |
| cana222 | 53.95 | -106.33 | 611 | 1671 | 1997 | PCMA | ITRDB |
| cana269 | 46.83 | -71.17 | 30 | 1540 | 2005 | THOC | ITRDB |
| cana283 | 62.57 | -114.35 | 209 | 1873 | 2005 | PIBN | ITRDB |
| cana284 | 62.52 | -114.35 | 189 | 1862 | 2005 | PIBN | ITRDB |
| cana285 | 62.50 | -114.35 | 194 | 1864 | 2005 | PIBN | ITRDB |
| cana286 | 62.48 | -114.37 | 211 | 1858 | 2005 | PIBN | ITRDB |
| cana287 | 62.48 | -114.42 | 208 | 1679 | 2005 | PIBN | ITRDB |
| cana288 | 62.42 | -114.42 | 175 | 1828 | 2005 | PIBN | ITRDB |
| cana289 | 62.47 | -114.30 | 191 | 1806 | 2005 | PIBN | ITRDB |
| cana290 | 62.60 | -114.13 | 195 | 1829 | 2005 | PIBN | ITRDB |
| cana291 | 62.55 | -113.85 | 197 | 1853 | 2005 | PIBN | ITRDB |
| cana292 | 62.52 | -113.82 | 199 | 1936 | 2005 | PIBN | ITRDB |
| cana293 | 62.55 | -113.87 | 214 | 1734 | 2005 | PIBN | ITRDB |
| cana294 | 62.50 | -113.43 | 228 | 1804 | 2005 | PIBN | ITRDB |
| cana327 | 52.18 | -116.43 | 1320 | 1576 | 2007 | PSME | ITRDB |
| cana328 | 52.05 | -116.40 | 1390 | 1018 | 2008 | PIFL | ITRDB |
| cana329 | 51.97 | -116.72 | 1423 | 1617 | 2007 | PIFL | ITRDB |
| cana330 | 52.00 | -116.45 | 1356 | 1062 | 2007 | PIFL | ITRDB |
| cana331 | 52.07 | -116.38 | 1420 | 1555 | 2008 | PSME | ITRDB |
| cana332 | 49.90 | -114.20 | 1661 | 1482 | 2004 | PSME | ITRDB |
| cana333 | 49.70 | -114.00 | 1395 | 1380 | 2004 | PSME | ITRDB |
| cana334 | 50.00 | -114.20 | 1677 | 1572 | 2004 | PSME | ITRDB |
| cana335 | 49.60 | -114.60 | 1384 | 1450 | 2004 | PIFL | ITRDB |
| cana338 | 51.30 | -114.70 | 1351 | 1341 | 2004 | PSME | ITRDB |
| cana339 | 49.90 | -114.10 | 1575 | 1525 | 2004 | PSME | ITRDB |
| cana340 | 62.00 | -128.30 | 500 | 1703 | 2001 | PCMA | ITRDB |
| cana341 | 62.03 | -128.27 | 1200 | 1666 | 2001 | PCMA | ITRDB |
| cana342 | 62.03 | -128.27 | 1200 | 1709 | 2001 | PCMA | ITRDB |
| cana343 | 49.90 | -114.40 | 1648 | 1618 | 2004 | PSME | ITRDB |
| cana344 | 56.63 | -119.62 | 516 | 1860 | 2006 | PIBN | ITRDB |
| cana347 | 49.90 | -114.20 | 1602 | 1509 | 2004 | PSME | ITRDB |
| cana348 | 54.75 | -115.62 | / | 1752 | 2004 | PCGL | ITRDB |
| cana349 | 54.75 | -115.62 | / | 1733 | 2004 | PIBN | ITRDB |
| cana351 | 52.68 | -118.33 | 1850 | 1104 | 1996 | PIAL | ITRDB |
| cana353 | 49.57 | -116.08 | 2025 | 932 | 1999 | PIAL | ITRDB |
| cana356 | 51.68 | -116.50 | 1950 | 1000 | 1999 | PIAL | ITRDB |
| cana364 | 59.20 | -129.65 | 1400 | 1767 | 2002 | ABLA | ITRDB |
| cana365 | 59.52 | -133.50 | 1400 | 1752 | 1999 | ABLA | ITRDB |
| cana366 | 60.60 | -135.07 | 1070 | 1764 | 2003 | ABLA | ITRDB |
| cana368 | 59.32 | -129.50 | 1360 | 1748 | 2002 | ABLA | ITRDB |
| cana369 | 59.18 | -129.67 | 1316 | 1738 | 2002 | ABLA | ITRDB |
| cana370 | 54.70 | -127.47 | 1400 | 1623 | 2002 | ABLA | ITRDB |
| cana372 | 60.53 | -128.85 | 1193 | 1496 | 2002 | ABLA | ITRDB |
| cana374 | 59.32 | -129.83 | 1343 | 1726 | 2002 | ABLA | ITRDB |
| cana375 | 60.57 | -133.08 | 1176 | 1796 | 2002 | ABLA | ITRDB |
| cana376 | 60.17 | -129.98 | 1355 | 1862 | 2002 | ABLA | ITRDB |
| cana377 | 60.10 | -130.67 | 1300 | 1737 | 1999 | ABLA | ITRDB |
| cana378 | 61.37 | -128.35 | 1450 | 1697 | 2002 | ABLA | ITRDB |
| cana379 | 59.72 | -133.37 | 1380 | 1756 | 1999 | ABLA | ITRDB |
| cana380 | 59.97 | -131.30 | 1150 | 1848 | 2002 | ABLA | ITRDB |
| cana381 | 54.77 | -127.27 | 1465 | 1670 | 2002 | ABLA | ITRDB |
| cana382 | 59.60 | -133.40 | 1350 | 1645 | 1999 | ABLA | ITRDB |
| cana383 | 63.83 | -137.38 | 1205 | 1765 | 2001 | ABLA | ITRDB |
| cana385 | 54.72 | -127.47 | 1830 | 1718 | 2002 | ABLA | ITRDB |
| cana386 | 55.38 | -127.03 | 1490 | 1742 | 2002 | ABLA | ITRDB |
| cana387 | 60.65 | -135.17 | 1160 | 1828 | 2003 | ABLA | ITRDB |
| cana395 | 51.65 | -116.68 | 2050 | 1519 | 1999 | PIAL | ITRDB |
| cana396 | 50.58 | -116.28 | 2200 | 1489 | 1999 | PIAL | ITRDB |
| cana397 | 50.20 | -115.22 | 2250 | 1720 | 1999 | PIAL | ITRDB |
| cana398 | 50.50 | -116.33 | 2125 | 1390 | 1997 | PIAL | ITRDB |
| cana399 | 49.72 | -115.58 | 2225 | 1477 | 1999 | PIAL | ITRDB |
| cana400 | 50.25 | -116.90 | 2100 | 1571 | 1999 | PIAL | ITRDB |
| cana401 | 51.10 | -117.15 | 2350 | 1715 | 1999 | PIAL | ITRDB |
| cana402 | 49.63 | -116.45 | 2250 | 1303 | 1999 | PIAL | ITRDB |
| cana403 | 50.63 | -117.37 | 1975 | 1670 | 1999 | PIAL | ITRDB |
| brit040 | 53.13 | -8.87 | 30 | 1841 | 1997 | QUSP | ITRDB |
| brit053 | 53.37 | -1.50 | 140 | 1759 | 2003 | QURO | ITRDB |
| brit054 | 53.35 | -6.32 | 52 | 1666 | 2008 | QUSP | ITRDB |
| brit055 | 54.55 | -5.93 | 25 | 1642 | 2004 | QUSP | ITRDB |
| brit056 | 54.30 | -5.83 | 62 | 1786 | 1997 | QUSP | ITRDB |
| brit057 | 54.22 | -5.93 | 62 | 1736 | 2006 | QUSP | ITRDB |
| cypr015 | 35.02 | 32.63 | 1050 | 1739 | 2002 | PIBR | ITRDB |
| cypr016 | 34.92 | 32.90 | 1550 | 1584 | 2002 | PIBR | ITRDB |
| cypr017 | 34.92 | 32.90 | 1640 | 1554 | 2002 | PINI | ITRDB |
| cypr018 | 34.93 | 32.87 | 1770 | 1379 | 2002 | PINI | ITRDB |
| cypr019 | 34.98 | 32.67 | 1400 | 1532 | 2002 | CDBR | ITRDB |
| czec003 | 48.67 | 14.70 | 785 | 1587 | 2010 | ABAL | ITRDB |
| czec004 | 48.67 | 14.70 | 785 | 1603 | 2010 | FASY | ITRDB |
| fran035 | 44.63 | 6.78 | 2300 | 1560 | 2000 | PICE | ITRDB |
| fran036 | 44.63 | 6.83 | 2350 | 1550 | 2000 | PICE | ITRDB |
| germ033 | 49.12 | 13.13 | 1420 | 1806 | 1997 | PCAB | ITRDB |
| germ034 | 48.92 | 12.58 | 370 | 1837 | 1998 | PCAB | ITRDB |
| germ035 | 48.92 | 12.13 | 390 | 1845 | 1996 | PCAB | ITRDB |
| germ036 | 48.97 | 11.98 | 425 | 1806 | 1996 | PCAB | ITRDB |
| germ037 | 49.03 | 12.55 | 680 | 1862 | 1996 | PCAB | ITRDB |
| germ038 | 49.05 | 12.82 | 970 | 1884 | 1996 | CAB | ITRDB |
| germ039 | 49.08 | 13.10 | 1208 | 1812 | 1996 | PCAB | ITRDB |
| germ040 | 49.10 | 13.33 | 1325 | 1540 | 1995 | PCAB | ITRDB |
| gree008 | 40.30 | 20.90 | 1500 | 1751 | 2003 | PINI | ITRDB |
| gree009 | 36.92 | 22.35 | 1400 | 1657 | 1999 | PINI | ITRDB |
| lith011 | 55.07 | 22.48 | 25 | 1878 | 2002 | QURO | ITRDB |
| lith012 | 55.97 | 21.08 | 12 | 1816 | 2002 | PISY | ITRDB |
| lith013 | 55.43 | 26.03 | 140 | 1890 | 2003 | PISY | ITRDB |
| norw009 | 69.08 | 17.22 | 150 | 1403 | 1997 | PISY | ITRDB |
| pola021 | 51.35 | 17.47 | 185 | 1890 | 2002 | ABAL | ITRDB |
| spai054 | 39.38 | -2.63 | 700 | 1924 | 1999 | PIPN | ITRDB |
| spai055 | 39.38 | -2.63 | 700 | 1920 | 1999 | PIPN | ITRDB |
| spai056 | 39.33 | -2.42 | 720 | 1882 | 1999 | PIPN | ITRDB |
| spai057 | 40.67 | -2.77 | 1055 | 1874 | 2001 | PIPN | ITRDB |
| spai059 | 39.28 | -1.35 | 705 | 1907 | 2001 | PIPN | ITRDB |
| swed314 | 57.85 | 14.63 | 295 | 1846 | 1996 | PISY | ITRDB |
| swed315 | 59.13 | 17.92 | 60 | 1800 | 1996 | PISY | ITRDB |
| swed316 | 60.13 | 16.08 | 135 | 1789 | 1997 | PISY | ITRDB |
| swed317 | 64.53 | 19.00 | 290 | 1724 | 1998 | ISY | ITRDB |
| swed318 | 63.25 | 12.50 | 525 | 1835 | 1996 | ISY | ITRDB |
| swed319 | 67.63 | 21.77 | 280 | 1656 | 1998 | ISY | ITRDB |
| swed320 | 59.15 | 18.00 | 75 | 1713 | 1996 | PISY | ITRDB |
| swed321 | 57.85 | 14.65 | 305 | 1844 | 1996 | PISY | ITRDB |
| swed322 | 63.25 | 12.42 | 530 | 1777 | 1996 | ISY | ITRDB |
| swed323 | 60.13 | 16.08 | 150 | 1723 | 1997 | PISY | ITRDB |
| swed324 | 62.33 | 18.48 | 250 | 1448 | 1998 | PISY | ITRDB |
| swed325 | 63.98 | 16.53 | 270 | 1520 | 1999 | ISY | ITRDB |
| swed326 | 63.12 | 13.33 | 700 | 1471 | 1998 | PISY | ITRDB |
| swed327 | 64.45 | 13.97 | 470 | 1500 | 2000 | PISY | ITRDB |
| swed328 | 59.18 | 18.27 | 60 | 1694 | 2000 | PISY | ITRDB |
| turk001 | 40.00 | 31.08 | 1400 | 1292 | 2001 | PINI | ITRDB |
| turk010 | 39.80 | 27.13 | 1200 | 1556 | 1999 | PINI | ITRDB |
| turk011 | 37.23 | 28.38 | 1200 | 1568 | 1999 | PINI | ITRDB |
| turk012 | 38.03 | 31.80 | 1400 | 1551 | 1998 | CDLI | ITRDB |
| turk013 | 37.42 | 30.28 | 1601 | 1511 | 2001 | PINI | ITRDB |
| turk014 | 37.42 | 30.30 | 1862 | 1246 | 2000 | JUEX | ITRDB |
| turk015 | 37.40 | 30.63 | 1156 | 1730 | 2000 | IBR | ITRDB |
| turk016 | 36.60 | 30.02 | 1853 | 1017 | 2006 | JUEX | ITRDB |
| turk017 | 36.60 | 30.02 | 1937 | 1449 | 2000 | CDLI | ITRDB |
| turk018 | 37.08 | 30.52 | 1047 | 1152 | 2000 | UEX | ITRDB |
| turk019 | 37.38 | 30.60 | 1469 | 1693 | 2000 | CDLI | ITRDB |
| turk020 | 36.65 | 32.20 | 1633 | 1586 | 2000 | PINI | ITRDB |
| turk021 | 36.65 | 32.18 | 1723 | 1628 | 2000 | CDLI | ITRDB |
| turk022 | 40.55 | 41.98 | 2100 | 1717 | 2001 | PISY | ITRDB |
| turk025 | 36.45 | 32.52 | 1770 | 1423 | 2003 | CDLI | ITRDB |
| turk030 | 39.28 | 28.93 | 1600 | 1771 | 2002 | PINI | ITRDB |
| turk031 | 37.63 | 35.43 | 1500 | 1475 | 2001 | PINI | ITRDB |
| turk032 | 37.03 | 30.47 | 700 | 1738 | 2001 | PIBR | ITRDB |
| turk036 | 37.15 | 30.52 | 1047 | 1694 | 2000 | IBR | ITRDB |
| mexi019 | 31.90 | -115.93 | 1580 | 1786 | 1995 | PIJE | ITRDB |
| mexi020 | 30.97 | -115.50 | 2400 | 1560 | 1995 | PIJE | ITRDB |
| ak014 | 58.05 | -152.70 | 10 | 1690 | 1996 | PCSI | ITRDB |
| ak021 | 60.00 | -141.68 | 650 | 1428 | 1995 | TSME | ITRDB |
| ak073 | 61.37 | -142.68 | 995 | 1546 | 1997 | PCGL | ITRDB |
| ak074 | 62.37 | -143.05 | 1167 | 1471 | 1997 | PCGL | ITRDB |
| ak075 | 61.15 | -142.08 | 1030 | 1580 | 1998 | PCGL | ITRDB |
| ak077 | 62.55 | -143.28 | 994 | 1559 | 1996 | PCGL | ITRDB |
| ak078 | 61.33 | -142.72 | 1040 | 1557 | 1997 | PCGL | ITRDB |
| ak086 | 60.48 | -154.33 | 550 | 1627 | 2003 | PCGL | ITRDB |
| ak087 | 60.50 | -153.88 | 580 | 1672 | 2003 | PCGL | ITRDB |
| ak088 | 60.97 | -153.92 | 400 | 1769 | 2003 | PCGL | ITRDB |
| ak089 | 60.65 | -154.02 | 580 | 1600 | 2003 | PCGL | ITRDB |
| ak091 | 61.05 | -147.10 | 90 | 1406 | 2002 | TSME | ITRDB |
| ak093 | 61.05 | -146.98 | 200 | 1472 | 1999 | TSME | ITRDB |
| ak096 | 61.05 | -146.98 | 200 | 616 | 2002 | TSME | ITRDB |
| ak102 | 58.43 | -135.60 | 530 | 1380 | 1999 | TSME | ITRDB |
| ak118 | 58.45 | -135.60 | 260 | 1566 | 2009 | CHNO | ITRDB |
| ak121 | 59.53 | -139.73 | 10 | 1403 | 1995 | PCSI | ITRDB |
| ak127 | 58.62 | -135.87 | 450 | 1535 | 2005 | PCSI | ITRDB |
| ak128 | 58.62 | -135.87 | 720 | 1562 | 2009 | TSME | ITRDB |
| ak129 | 58.62 | -135.87 | 770 | 1618 | 2009 | TSME | ITRDB |
| ak130 | 58.30 | -134.38 | 540 | 1557 | 1999 | TSME | ITRDB |
| ak131 | 58.38 | -134.43 | 220 | 1450 | 2010 | TSME | ITRDB |
| al002 | 31.02 | -87.25 | 63 | 1814 | 1995 | PIPA | ITRDB |
| az556 | 31.45 | -110.35 | 1750 | 1630 | 1995 | PSME | ITRDB |
| az557 | 32.45 | -110.78 | 2550 | 1321 | 1998 | PSME | ITRDB |
| az560 | 36.13 | -111.88 | 2800 | 1800 | 1995 | PIPO | ITRDB |
| az562 | 36.10 | -111.87 | 2200 | 1757 | 1995 | PIPO | ITRDB |
| ca598 | 36.07 | -121.57 | 625 | 1830 | 1995 | PIPO | ITRDB |
| ca600 | 33.13 | -116.60 | 1465 | 1886 | 1995 | PICL | ITRDB |
| ca601 | 33.13 | -116.60 | 1465 | 1808 | 1995 | PSMA | ITRDB |
| ca603 | 37.92 | -119.23 | 3300 | 1430 | 1996 | PIAL | ITRDB |
| ca604 | 37.92 | -119.28 | 3300 | 1520 | 1996 | PIAL | ITRDB |
| ca605 | 37.87 | -119.28 | 3300 | 885 | 1996 | PIAL | ITRDB |
| ca606 | 37.83 | -119.22 | 3300 | 800 | 1996 | PIAL | ITRDB |
| ca608 | 38.02 | -122.80 | 120 | 1760 | 1997 | PSME | ITRDB |
| ca609 | 34.12 | -116.80 | 2890 | 1560 | 1995 | PIJE | ITRDB |
| ca610 | 32.87 | -116.42 | 1800 | 1660 | 1995 | PIJE | ITRDB |
| ca613 | 33.73 | -117.55 | 1200 | 1660 | 1995 | PSMA | ITRDB |
| ca614 | 35.30 | -120.27 | 561 | 1455 | 2004 | QUDG | ITRDB |
| ca615 | 39.02 | -122.82 | 426 | 1620 | 2004 | QUDG | ITRDB |
| ca616 | 37.72 | -120.42 | 274 | 1531 | 2005 | QUDG | ITRDB |
| ca617 | 39.82 | -123.07 | 610 | 1535 | 1996 | QUDG | ITRDB |
| ca618 | 39.52 | -121.43 | 305 | 1572 | 2004 | QUDG | ITRDB |
| ca619 | 40.27 | -121.85 | 853 | 1393 | 2004 | QUDG | ITRDB |
| ca620 | 38.77 | -121.10 | 167 | 1603 | 2003 | QUDG | ITRDB |
| ca621 | 35.52 | -118.67 | 670 | 1585 | 2003 | QUDG | ITRDB |
| ca623 | 37.87 | -121.95 | 185 | 1582 | 2004 | QUDG | ITRDB |
| ca624 | 37.87 | -121.95 | 245 | 1621 | 1996 | QULO | ITRDB |
| ca625 | 37.05 | -121.35 | 396 | 1510 | 2003 | QUDG | ITRDB |
| ca626 | 36.47 | -121.18 | 350 | 1577 | 2003 | QUDG | ITRDB |
| ca627 | 37.03 | -119.67 | 213 | 1710 | 1996 | QUDG | ITRDB |
| ca628 | 40.15 | -120.60 | 1480 | 1450 | 1998 | PIPO | ITRDB |
| ca629 | 41.45 | -120.90 | 1302 | 1152 | 1998 | JUOC | ITRDB |
| ca630 | 38.70 | -120.00 | 2591 | -420 | 1999 | JUOC | ITRDB |
| ca631 | 39.52 | -120.55 | 1921 | 930 | 1999 | JUOC | ITRDB |
| ca632 | 39.55 | -120.20 | 2268 | 1010 | 1999 | JUOC | ITRDB |
| ca633 | 37.75 | -118.68 | 2990 | 680 | 2000 | PIFL | ITRDB |
| ca635 | 37.63 | -121.77 | 149 | 1896 | 2003 | QULO | ITRDB |
| ca641 | 37.87 | -119.37 | 2630 | 1700 | 2005 | PICO | ITRDB |
| ca642 | 37.88 | -119.37 | 2626 | 1700 | 2005 | PICO | ITRDB |
| ca643 | 37.87 | -119.37 | 2615 | 1700 | 2005 | PICO | ITRDB |
| ca645 | 37.67 | -122.30 | 475 | 1697 | 2003 | QUDG | ITRDB |
| ca646 | 36.82 | -121.47 | 1067 | 1379 | 2003 | QUDG | ITRDB |
| ca647 | 36.57 | -120.87 | 1219 | 1409 | 2003 | QUDG | ITRDB |
| ca648 | 40.88 | -120.03 | 396 | 1582 | 2004 | QUDG | ITRDB |
| ca649 | 39.35 | -122.73 | 468 | 1546 | 2004 | QUDG | ITRDB |
| ca650 | 40.47 | -122.75 | 421 | 1649 | 2004 | QUDG | ITRDB |
| ca651 | 36.48 | -119.30 | 1132 | 1596 | 2003 | QUDG | ITRDB |
| ca652 | 40.42 | -122.63 | 218 | 1519 | 2004 | QUDG | ITRDB |
| ca653 | 36.52 | -119.12 | 1237 | 1448 | 2003 | QUDG | ITRDB |
| ca654 | 40.15 | -122.05 | 143 | 1601 | 2005 | QUDG | ITRDB |
| ca655 | 38.00 | -121.02 | 457 | 1695 | 2004 | QUDG | ITRDB |
| ca656 | 35.23 | -120.00 | 1036 | 1293 | 2003 | QUDG | ITRDB |
| ca657 | 36.65 | -121.92 | 755 | 1460 | 2004 | QUDG | ITRDB |
| ca658 | 36.20 | -121.77 | 646 | 1494 | 2003 | QUDG | ITRDB |
| ca659 | 36.92 | -119.48 | 701 | 1494 | 2004 | QUDG | ITRDB |
| ca660 | 35.53 | -119.40 | 1051 | 1333 | 2004 | QUDG | ITRDB |
| ca661 | 40.40 | -123.02 | 342 | 1532 | 2004 | QUDG | ITRDB |
| ca662 | 36.15 | -120.43 | 558 | 1538 | 2004 | QUDG | ITRDB |
| ca663 | 39.12 | -122.45 | 180 | 1534 | 2004 | QUDG | ITRDB |
| ca664 | 37.15 | -120.25 | 345 | 1557 | 2004 | QUDG | ITRDB |
| ca665 | 38.45 | -120.63 | 1194 | 1408 | 2004 | QUDG | ITRDB |
| ca671 | 37.62 | -119.02 | 2770 | 1610 | 2009 | PICO | ITRDB |
| ca674 | 40.10 | -120.63 | 1385 | 1450 | 2010 | PIPO | ITRDB |
| ca675 | 41.83 | -120.88 | 1508 | 1152 | 2010 | JUOC | ITRDB |
| ca676 | 41.67 | -120.98 | 1513 | 1357 | 2010 | PIPO | ITRDB |
| ca677 | 39.57 | -120.28 | 1688 | 1415 | 2010 | PIJE | ITRDB |
| ca678 | 37.95 | -119.15 | 2499 | 1304 | 2010 | PIJE | ITRDB |
| ca679 | 42.03 | -120.57 | 1645 | 1421 | 2010 | PIPO | ITRDB |
| ca686 | 37.55 | -121.85 | 89 | 1700 | 2010 | PLRA | ITRDB |
| ca687 | 37.55 | -121.85 | 89 | 1697 | 2010 | QULO | ITRDB |
| co555 | 37.78 | -105.50 | 2530 | 1260 | 1995 | PIPO | ITRDB |
| co556 | 37.72 | -105.47 | 2865 | 1035 | 1995 | PIFL | ITRDB |
| co557 | 37.78 | -106.82 | 2877 | 1654 | 1997 | PIPO | ITRDB |
| co558 | 38.10 | -106.37 | 2621 | 1605 | 1997 | PIPO | ITRDB |
| co559 | 37.38 | -106.28 | 2658 | 1600 | 1997 | PIPO | ITRDB |
| co560 | 37.63 | -106.68 | 2560 | 1675 | 1997 | PIPO | ITRDB |
| co563 | 40.42 | -105.57 | 2816 | 1698 | 2001 | PICO | ITRDB |
| co564 | 39.50 | -104.22 | 1800 | 1709 | 1997 | PIPO | ITRDB |
| co565 | 39.38 | -104.20 | 1850 | 1779 | 1998 | PIPO | ITRDB |
| co566 | 39.07 | -104.43 | 2170 | 1534 | 1998 | PSME | ITRDB |
| co567 | 37.50 | -103.53 | 1600 | 1743 | 1997 | PIED | ITRDB |
| co568 | 37.23 | -103.25 | 1650 | 1628 | 1998 | PIPO | ITRDB |
| co569 | 37.10 | -103.62 | 2060 | 1464 | 1997 | PIPO | ITRDB |
| co570 | 37.07 | -103.27 | 1580 | 1460 | 1998 | PIPO | ITRDB |
| co571 | 37.07 | -103.27 | 1580 | 1837 | 1998 | PIED | ITRDB |
| co572 | 40.32 | -105.55 | 2800 | 894 | 1998 | PSME | ITRDB |
| co579 | 39.97 | -106.52 | 2194 | 1320 | 2002 | PIED | ITRDB |
| co581 | 40.75 | -106.85 | 2377 | 1539 | 1999 | PSME | ITRDB |
| co582 | 38.67 | -108.35 | 1737 | 1569 | 1999 | PIED | ITRDB |
| co583 | 39.72 | -106.98 | 2210 | 1402 | 2002 | PIED | ITRDB |
| co584 | 39.70 | -106.73 | 2164 | 1336 | 1999 | PIED | ITRDB |
| co585 | 41.15 | -106.78 | 2500 | 1380 | 2001 | PSME | ITRDB |
| co588 | 37.47 | -106.30 | 2835 | 1632 | 2004 | PIPO | ITRDB |
| co589 | 38.73 | -106.80 | 2926 | 1319 | 1999 | PSME | ITRDB |
| co590 | 40.67 | -105.52 | 2301 | 1394 | 2002 | PIPO | ITRDB |
| co591 | 40.98 | -105.67 | 2650 | 1423 | 2001 | PIPO | ITRDB |
| co592 | 40.42 | -105.28 | 2012 | 1550 | 2000 | PSME | ITRDB |
| co593 | 38.08 | -107.00 | 2895 | 1372 | 2002 | PSME | ITRDB |
| co594 | 38.25 | -106.67 | 2835 | 1437 | 2002 | PIPO | ITRDB |
| co595 | 39.83 | -108.20 | 2050 | 1168 | 2001 | PIED | ITRDB |
| co596 | 39.93 | -105.30 | 2002 | 1545 | 2003 | PIPO | ITRDB |
| co597 | 39.60 | -105.90 | 2880 | 1372 | 2002 | PSME | ITRDB |
| co598 | 40.37 | -105.58 | 2652 | 1547 | 2000 | PIPO | ITRDB |
| co599 | 39.60 | -108.80 | 2591 | 1382 | 2000 | PSME | ITRDB |
| co600 | 38.25 | -108.33 | 1996 | 1536 | 2000 | PIED | ITRDB |
| co601 | 39.38 | -105.17 | 2103 | 1401 | 1997 | PIPO | ITRDB |
| co602 | 38.87 | -105.43 | 2743 | 1507 | 1997 | PIPO | ITRDB |
| co603 | 39.93 | -105.30 | 2002 | 1541 | 2002 | PSME | ITRDB |
| co604 | 39.85 | -106.23 | 2514 | 1378 | 2000 | PSME | ITRDB |
| co605 | 38.60 | -107.58 | 2271 | 1385 | 2002 | PIED | ITRDB |
| co606 | 40.07 | -106.13 | 2499 | 1571 | 1999 | PSME | ITRDB |
| co607 | 40.13 | -105.42 | 2469 | 1364 | 2000 | PIPO | ITRDB |
| co608 | 39.68 | -105.20 | 1965 | 1487 | 2003 | PIPO | ITRDB |
| co616 | 40.78 | -108.97 | 2133 | 1270 | 2000 | PIED | ITRDB |
| co617 | 38.80 | -106.23 | 2956 | 1169 | 2002 | PSME | ITRDB |
| co618 | 38.80 | -106.22 | 2774 | 1462 | 1999 | PIED | ITRDB |
| co620 | 38.53 | -107.22 | 2835 | 1525 | 2000 | PSME | ITRDB |
| co621 | 39.67 | -107.88 | 2073 | 1335 | 2000 | PIED | ITRDB |
| co622 | 40.72 | -105.58 | 2499 | 1436 | 2002 | PIPO | ITRDB |
| co623 | 38.32 | -107.20 | 2700 | 1511 | 2000 | PIPO | ITRDB |
| co624 | 38.40 | -106.43 | 2621 | 1275 | 2000 | PSME | ITRDB |
| co625 | 37.67 | -106.65 | 2591 | 1566 | 2002 | PIPO | ITRDB |
| co626 | 38.02 | -108.92 | 2000 | 1490 | 2002 | PIED | ITRDB |
| co627 | 38.53 | -107.32 | 2417 | 1541 | 1999 | PIPO | ITRDB |
| co628 | 38.33 | -105.27 | 2465 | 1480 | 1997 | PSME | ITRDB |
| co629 | 38.83 | -108.57 | 2225 | 1296 | 2000 | PIED | ITRDB |
| co630 | 40.03 | -106.07 | 2865 | 1454 | 1999 | PSME | ITRDB |
| co631 | 39.02 | -108.23 | 2636 | 1146 | 2002 | PIED | ITRDB |
| co632 | 37.90 | -105.15 | 2690 | 1336 | 1997 | PSME | ITRDB |
| co636 | 37.82 | -106.38 | 2667 | 1508 | 2002 | PIED | ITRDB |
| co637 | 37.55 | -105.57 | 2575 | 1450 | 2003 | PIED | ITRDB |
| co638 | 37.22 | -107.85 | 2012 | 1566 | 2002 | PIED | ITRDB |
| co639 | 39.02 | -105.37 | 2440 | 1620 | 2003 | PIPO | ITRDB |
| co640 | 38.40 | -105.30 | 1900 | 1577 | 2003 | PIED | ITRDB |
| co641 | 39.80 | -105.25 | 1920 | 1566 | 2003 | PIPO | ITRDB |
| ga006 | 31.22 | -84.48 | 43 | 1802 | 1995 | PIPA | ITRDB |
| ga007 | 31.22 | -84.48 | 43 | 1911 | 1995 | PIPA | ITRDB |
| ga008 | 31.22 | -84.48 | 43 | 1871 | 1995 | PIPA | ITRDB |
| ga012 | 34.67 | -84.27 | 784 | 1537 | 2009 | LITU | ITRDB |
| ga013 | 34.47 | -84.32 | 730 | 1646 | 2009 | QUSP | ITRDB |
| ga014 | 34.77 | -84.30 | 777 | 1689 | 2009 | TSCA | ITRDB |
| ga015 | 30.85 | -83.97 | 61 | 1661 | 2003 | PIPA | ITRDB |
| ga016 | 30.33 | -85.25 | 344 | 1795 | 2003 | PIPA | ITRDB |
| ga017 | 32.85 | -84.48 | 262 | 1649 | 2002 | PIPA | ITRDB |
| ga018 | 32.25 | -85.20 | 262 | 1730 | 2001 | PIEC | ITRDB |
| ga019 | 32.48 | -84.33 | 198 | 1762 | 2002 | QUST | ITRDB |
| ga022 | 32.83 | -84.83 | 366 | 1794 | 2002 | QUPR | ITRDB |
| id013 | 44.62 | -114.50 | 2820 | 1305 | 1997 | PCEN | ITRDB |
| id015 | 43.75 | -116.10 | 1825 | 1488 | 2011 | PIPO | ITRDB |
| mt109 | 45.17 | -109.52 | 3208 | 1660 | 1999 | PCEN | ITRDB |
| mt110 | 45.30 | -111.33 | 2500 | 819 | 2000 | PSME | ITRDB |
| mt111 | 45.30 | -111.33 | 2500 | 500 | 2000 | PIFL | ITRDB |
| mt112 | 45.30 | -111.32 | 2500 | 470 | 1998 | PIFL | ITRDB |
| mt113 | 45.30 | -111.32 | 2500 | 470 | 1998 | PIFL | ITRDB |
| mt114 | 45.30 | -111.32 | 2500 | 952 | 1998 | PSME | ITRDB |
| mt116 | 46.28 | -113.15 | 2645 | 999 | 1998 | LALY | ITRDB |
| mt117 | 48.72 | -113.65 | 2150 | 1766 | 2006 | ABLA | ITRDB |
| mt127 | 45.00 | -110.68 | 1865 | -413 | 2008 | JUSC | ITRDB |
| nc012 | 35.35 | -83.92 | 250 | 1672 | 1997 | LITU | ITRDB |
| nc013 | 35.35 | -83.92 | 250 | 1784 | 1997 | TSCA | ITRDB |
| nc014 | 35.60 | -83.42 | 1372 | 1771 | 1997 | QURU | ITRDB |
| nc022 | 35.50 | -82.63 | 790 | 1716 | 1996 | QUSP | ITRDB |
| nc023 | 35.03 | -83.27 | 1280 | 1599 | 2003 | QUAL | ITRDB |
| ne005 | 42.70 | -100.87 | 810 | 1728 | 1998 | PIPO | ITRDB |
| ne006 | 42.67 | -99.72 | 670 | 1732 | 1997 | PIPO | ITRDB |
| ne007 | 41.52 | -103.93 | 1530 | 1489 | 1997 | PIPO | ITRDB |
| ne008 | 42.63 | -103.25 | 1280 | 1567 | 1997 | PIPO | ITRDB |
| nh005 | 43.80 | -71.83 | 300 | 1690 | 2008 | PIRE | ITRDB |
| nm574 | 36.80 | -103.98 | 2020 | 1613 | 1998 | PIPO | ITRDB |
| nm575 | 36.77 | -103.97 | 2240 | 1712 | 1998 | PIPO | ITRDB |
| nm576 | 36.77 | -103.95 | 2380 | 1626 | 1998 | PIED | ITRDB |
| nm577 | 36.07 | -104.35 | 1710 | 1595 | 1998 | PIPO | ITRDB |
| nm580 | 34.97 | -108.18 | 2375 | -136 | 2004 | PSME | ITRDB |
| nm581 | 36.53 | -106.02 | 2300 | 1520 | 2003 | PIED | ITRDB |
| nm582 | 36.62 | -105.98 | 2500 | 1575 | 2003 | PIPO | ITRDB |
| nm583 | 36.35 | 106.52 | 2059 | 1295 | 2007 | PSME | ITRDB |
| nm584 | 36.28 | 106.62 | 2525 | 644 | 2007 | PSME | ITRDB |
| nm585 | 35.92 | 106.67 | 2597 | 1568 | 2007 | PISF | ITRDB |
| nm586 | 35.92 | 106.68 | 2561 | 1298 | 2007 | PSME | ITRDB |
| nm587 | 35.88 | 106.68 | 2529 | 1304 | 2007 | PSME | ITRDB |
| nv518 | 41.30 | -118.43 | 2097 | 975 | 1998 | JUOC | ITRDB |
| nv519 | 37.87 | -118.33 | 3140 | 990 | 2000 | PIFL | ITRDB |
| oh004 | 39.45 | -82.15 | 314 | 1856 | 1995 | QUAL | ITRDB |
| oh005 | 39.45 | -82.15 | 314 | 1681 | 1995 | QUAL | ITRDB |
| oh006 | 39.98 | -81.00 | 384 | 1625 | 1998 | QUAL | ITRDB |
| or046 | 43.75 | -121.65 | 1320 | 1529 | 1995 | PIPO | ITRDB |
| or050 | 43.62 | -121.30 | 1550 | 1747 | 1995 | PIPO | ITRDB |
| or051 | 43.47 | -121.40 | 1420 | 1574 | 1995 | PIPO | ITRDB |
| or052 | 43.32 | -121.75 | 1420 | 1419 | 1995 | PIPO | ITRDB |
| or053 | 43.23 | -121.65 | 1670 | 1639 | 1995 | PIPO | ITRDB |
| or054 | 43.08 | -121.95 | 1510 | 1513 | 1995 | PIPO | ITRDB |
| or055 | 42.92 | -121.53 | 1490 | 1423 | 1995 | PIPO | ITRDB |
| or056 | 42.93 | -121.62 | 1550 | 1570 | 1995 | PIPO | ITRDB |
| or057 | 42.75 | -121.52 | 1550 | 1442 | 1995 | PIPO | ITRDB |
| or059 | 42.63 | -121.53 | 2020 | 1653 | 1995 | PIPO | ITRDB |
| or060 | 43.58 | -120.45 | 1494 | 870 | 1996 | JUOC | ITRDB |
| or062 | 43.18 | -120.90 | 1418 | 530 | 1996 | JUOC | ITRDB |
| or063 | 42.67 | -118.92 | 1460 | 1017 | 1998 | JUOC | ITRDB |
| or082 | 44.70 | -118.55 | 2295 | 1740 | 2002 | PIAL | ITRDB |
| or083 | 44.70 | -118.55 | 2295 | 1892 | 2002 | PICO | ITRDB |
| or084 | 44.70 | -118.55 | 2295 | 1865 | 2002 | ABLA | ITRDB |
| or085 | 45.50 | -121.42 | 1002 | 1602 | 1999 | PIPO | ITRDB |
| or089 | 44.57 | -121.27 | 730 | 1733 | 1996 | JUOC | ITRDB |
| or092 | 43.17 | -120.88 | 1428 | 530 | 2010 | JUOC | ITRDB |
| or093 | 43.70 | -120.47 | 1475 | 870 | 2010 | JUOC | ITRDB |
| or094 | 43.95 | -121.05 | 1146 | 830 | 2010 | JUOC | ITRDB |
| or095 | 43.15 | -119.80 | 1514 | 1337 | 2010 | JUOC | ITRDB |
| sd020 | 45.28 | -100.73 | 501 | 1840 | 2006 | PPDE | ITRDB |
| tn016 | 35.60 | -83.08 | 1065 | 1736 | 1995 | LITU | ITRDB |
| tn018 | 35.75 | -83.23 | 233 | 1765 | 1995 | TSCA | ITRDB |
| tn019 | 35.75 | -83.23 | 218 | 1793 | 1995 | TSCA | ITRDB |
| tn020 | 35.62 | -85.43 | 700 | 1743 | 1997 | QUVE | ITRDB |
| tn021 | 35.62 | -85.43 | 700 | 1750 | 1997 | QUPR | ITRDB |
| tn023 | 35.67 | -83.50 | 825 | 1825 | 1995 | LITU | ITRDB |
| tn024 | 35.67 | -83.50 | 825 | 1685 | 1995 | TSCA | ITRDB |
| tn026 | 35.75 | -83.23 | 925 | 1895 | 1995 | TSCA | ITRDB |
| tn027 | 35.67 | -82.38 | 1005 | 1698 | 1997 | LITU | ITRDB |
| tx009 | 32.98 | -99.18 | 427 | 1681 | 1995 | QUST | ITRDB |
| tx018 | 30.93 | -96.80 | 106 | 1668 | 1995 | QUST | ITRDB |
| tx051 | 33.40 | -98.08 | 275 | 1793 | 2006 | QUST | ITRDB |
| tx053 | 30.48 | -98.15 | 230 | 1423 | 2009 | TADI | ITRDB |
| va027 | 37.55 | -79.07 | 230 | 1749 | 1998 | QUAL | ITRDB |
| wi006 | 42.67 | -87.90 | 217 | 1807 | 2000 | QUAL | ITRDB |
| wv006 | 37.52 | -80.98 | 670 | 1858 | 2012 | TSCA | ITRDB |
| wv007 | 37.98 | -82.37 | 250 | 1756 | 2012 | TSCA | ITRDB |
| wy026 | 41.87 | -110.80 | 2225 | 1480 | 1998 | PIFL | ITRDB |
| wy027 | 44.98 | -110.67 | 2179 | 1168 | 1999 | PSME | ITRDB |
| wy031 | 41.15 | -106.78 | 2500 | 1380 | 2001 | PSME | ITRDB |
| wy032 | 41.15 | -105.37 | 2500 | 1511 | 2001 | PIPO | ITRDB |
| wy033 | 44.75 | -110.25 | 2542 | 1390 | 2005 | PIAL | ITRDB |
| wy035 | 44.62 | -110.43 | 2400 | 1900 | 2008 | PICO | ITRDB |
| wy036 | 44.60 | -110.40 | 2378 | 1734 | 2009 | PICO | ITRDB |
| wy046 | 44.73 | -109.90 | 2961 | 1440 | 2012 | PCEN | ITRDB |
| wy047 | 44.57 | -110.38 | 2395 | 1758 | 2010 | PICO | ITRDB |
| wy048 | 44.70 | -110.50 | 2349 | 1680 | 2010 | PICO | ITRDB |
| wy050 | 44.80 | -110.43 | 3030 | 937 | 1998 | PIAL | ITRDB |
| wy051 | 44.73 | -110.72 | 2675 | 1597 | 2009 | PICO | ITRDB |
| CHTS001 | 43.82 | 93.31 | 2600 | 1910 | 2000 | LASI | This study |
| CHTS002 | 43.82 | 93.34 | 2660 | 1905 | 2000 | LASI | This study |
| CHWS001 | 44.12 | 84.60 | 2135 | 1915 | 2005 | PCSH | This study |
| CHWS002 | 44.13 | 84.61 | 1838 | 1920 | 2005 | PCSH | This study |
| CHTC001 | 43.93 | 88.11 | 1740 | 1943 | 2005 | PCSH | This study |
| CHJM001 | 43.89 | 88.67 | 1886 | 1935 | 2005 | PCSH | This study |
| CHJM002 | 43.90 | 88.68 | 2005 | 1921 | 2005 | PCSH | This study |
| CHJH001 | 44.35 | 83.13 | 1770 | 1921 | 2006 | PCSH | This study |
| CHJH002 | 44.32 | 83.13 | 2200 | 1896 | 2006 | PCSH | This study |
| CHWM001 | 43.28 | 87.18 | 2100 | 1906 | 2005 | PCSH | This study |
| CHWM002 | 43.43 | 87.24 | 1532 | 1924 | 2005 | PCSH | This study |
| CHST001 | 44.47 | 81.44 | 2454 | 1905 | 2005 | PCSH | This study |
| CHST002 | 44.47 | 81.48 | 2000 | 1913 | 2005 | PCSH | This study |
| CHGQ001 | 43.24 | 116.39 | 1363 | 1843 | 2004 | PITB | Liang et al., 2007 |
| CHYI001 | 43.09 | 116.60 | 1331 | 1820 | 2004 | PITB | Liang et al., 2007 |
| CHSS001 | 43.90 | 116.96 | 1285 | 1833 | 2004 | PITB | Liang et al., 2007 |
| CHXS001 | 42.90 | 116.96 | 1250 | 1880 | 2004 | PITB | Liang et al., 2008 |
| CHBY001 | 42.99 | 117.05 | 1329 | 1848 | 2004 | PITB | Liang et al., 2007 |
| CHLI001 | 43.63 | 117.72 | 1197 | 1833 | 2004 | PITB | Liang et al., 2008 |
| CHTB001 | 33.92 | 107.78 | 3190 | 1899 | 2006 | ABCH | This study |
| CHTB002 | 33.91 | 107.79 | 3121 | 1907 | 2006 | ABCH | This study |
| CHTB003 | 33.91 | 107.79 | 3075 | 1908 | 2006 | ABCH | This study |
| CHTB004 | 33.94 | 107.77 | 3295 | 1886 | 2006 | *Larix chinensis* | This study |
| CHTB005 | 33.93 | 107.78 | 3190 | 1912 | 2006 | *Larix chinensis* | This study |
| CHTB006 | 33.92 | 107.79 | 3123 | 1892 | 2006 | *Larix chinensis* | This study |

Note:

* The site names are consistent with those in the records of ITRDB (https://www.ncdc.noaa.gov/data-access/paleoclimatology-data/datasets/tree-ring), apart from those provided by our group and those from Liang *et al*. (2007, 2008).

ǂ The abbreviations used for the species sampled are consistent with those of ITRDB. Names of species which are not recorded in ITRDB are shown in full.

† ITRDB: international tree ring data bank (https://www.ncdc.noaa.gov/data-access/paleoclimatology-data/datasets/tree-ring).

Supplementary Figure 1.


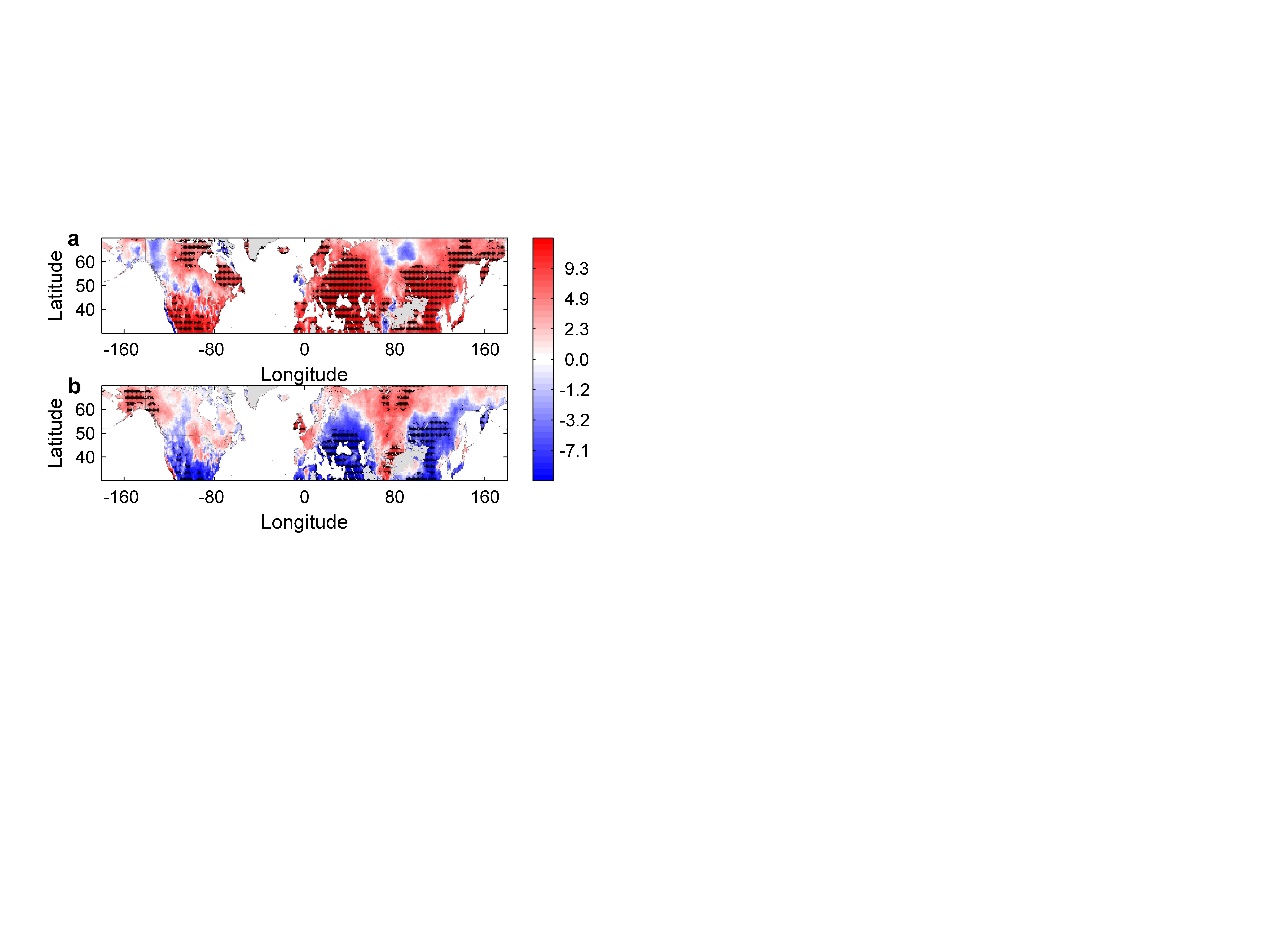


Supplementary Figure 1. Spatial pattern in linear trends in the accumulated temperature exposure above (a) and below (b) the 95^th^ percentile of daily temperature distribution for growing seasons during 1982-2012. Stratified regions indicate statistical significance at *p* < 0.05. Regions with multi-year mean Normalized Difference Vegetation Index (NDVI) values < 0.1 during 1982-2012 were discarded from our analyses (blank regions).

Supplementary Figure 2


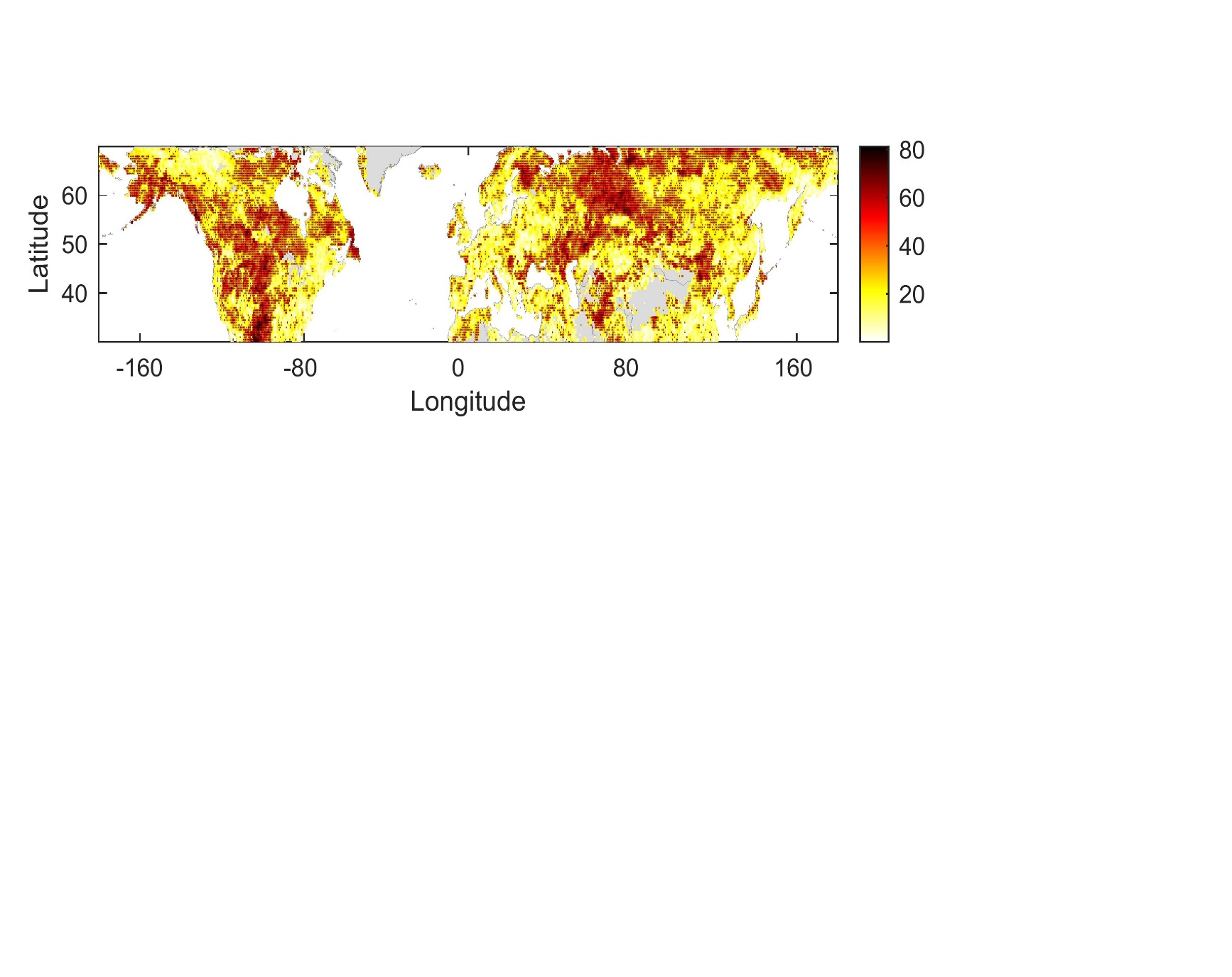


Supplementary Figure 2. Spatial pattern in the goodness (%) of fit of the ridge regression fitted between the mean growing season (April−October) Normalized Difference Vegetation Index (NDVI) and total growing-season precipitation, mean growing-season solar radiation, mean growing-season temperature and accumulated temperature exposure above and below the 95^th^ percentile of daily temperature distribution for growing seasons during 1982-2012. Stratified regions indicate statistical significance at *p* < 0.05. Regions with multi-year mean NDVI values < 0.1 during 1982-2012 were discarded from our analyses (blank regions).

Supplementary Figure 3.


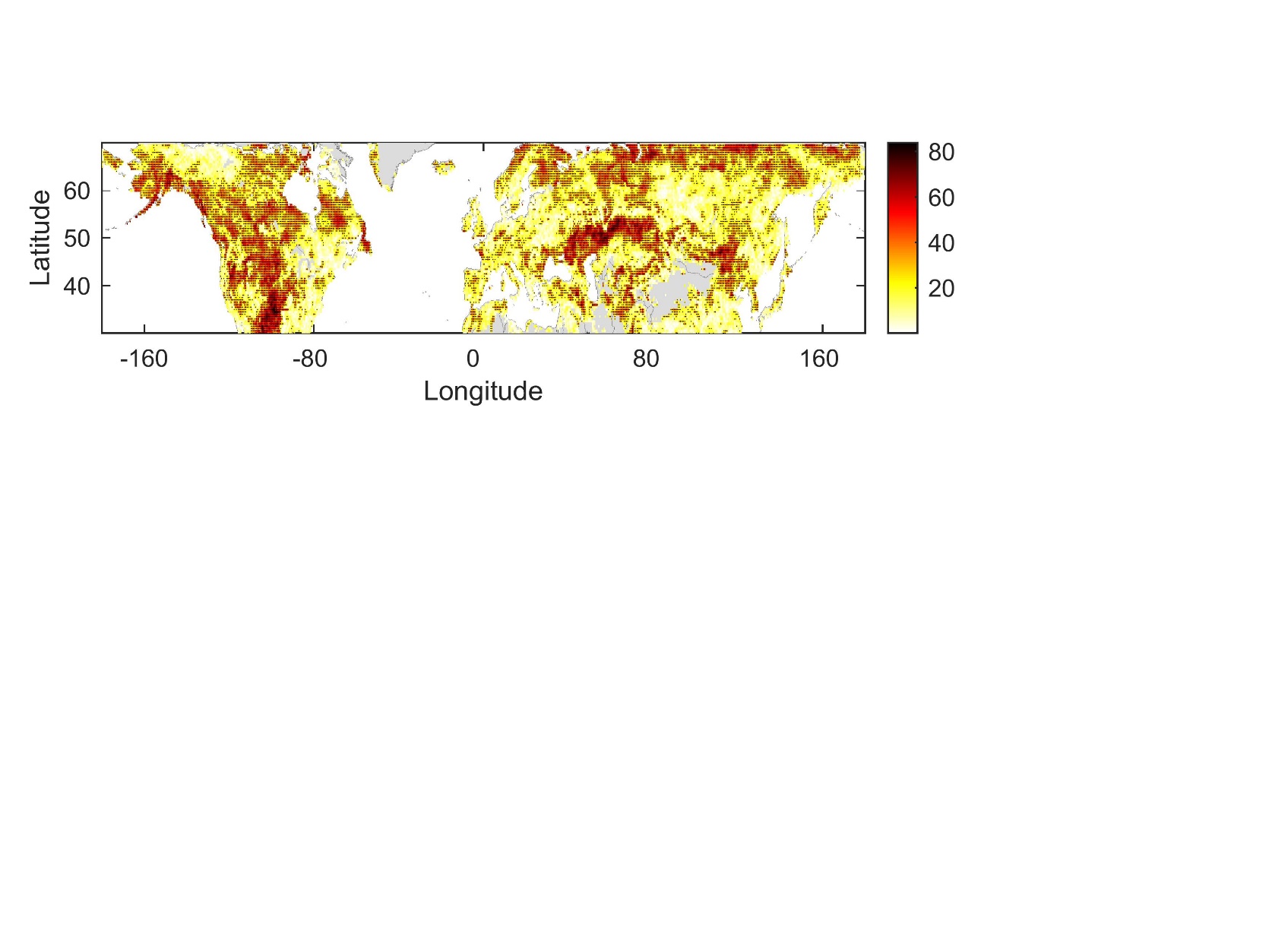


Supplementary Figure 3. Spatial pattern in the goodness (%) of fit of the ridge regression fitted between the mean growing-season (April-October) Normalized Difference Vegetation Index (NDVI) and total growing-season precipitation, and cumulative temperature exposures above and below the 95^th^ percentile of daily temperature distribution for growing seasons during 1982-2012. Stratified regions indicate statistical significance at *p* < 0.05. Regions with multi-year mean NDVI values < 0.1 during 1982-2012 were discarded from our analyses (blank regions).

Supplementary Figure 4.


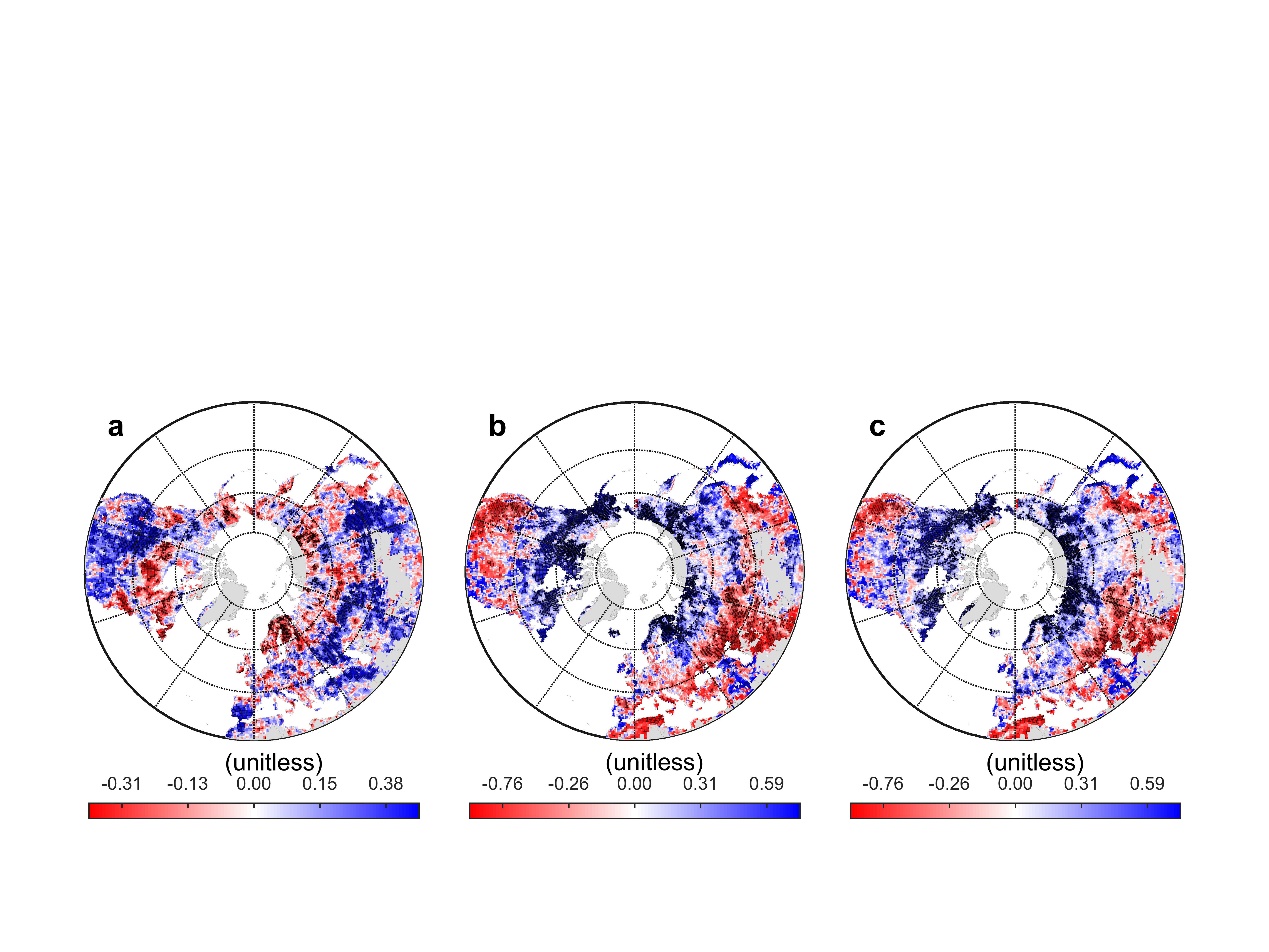


**Supplementary Figure 4. Spatial patterns of the standard regression coefficients between the interannual variations of the mean growing season (April–October) Normalized Difference Vegetation Index (NDVI_GS_) and climate.** A ridge regression was performed between NDVI_GS_ and total growing season precipitation (a), accumulated temperature exposure above (b, TE_H_) and below (c, TE_L_) the 90^th^ percentile of the daily temperature distribution for the growing seasons during the period 1982–2012. Stratified regions are statistically significant at *p* < 0.05. Regions with multi-year mean NDVI values < 0.1 during 1982–2012 were discarded from our analyses (blank regions).

Supplementary Figure 5.


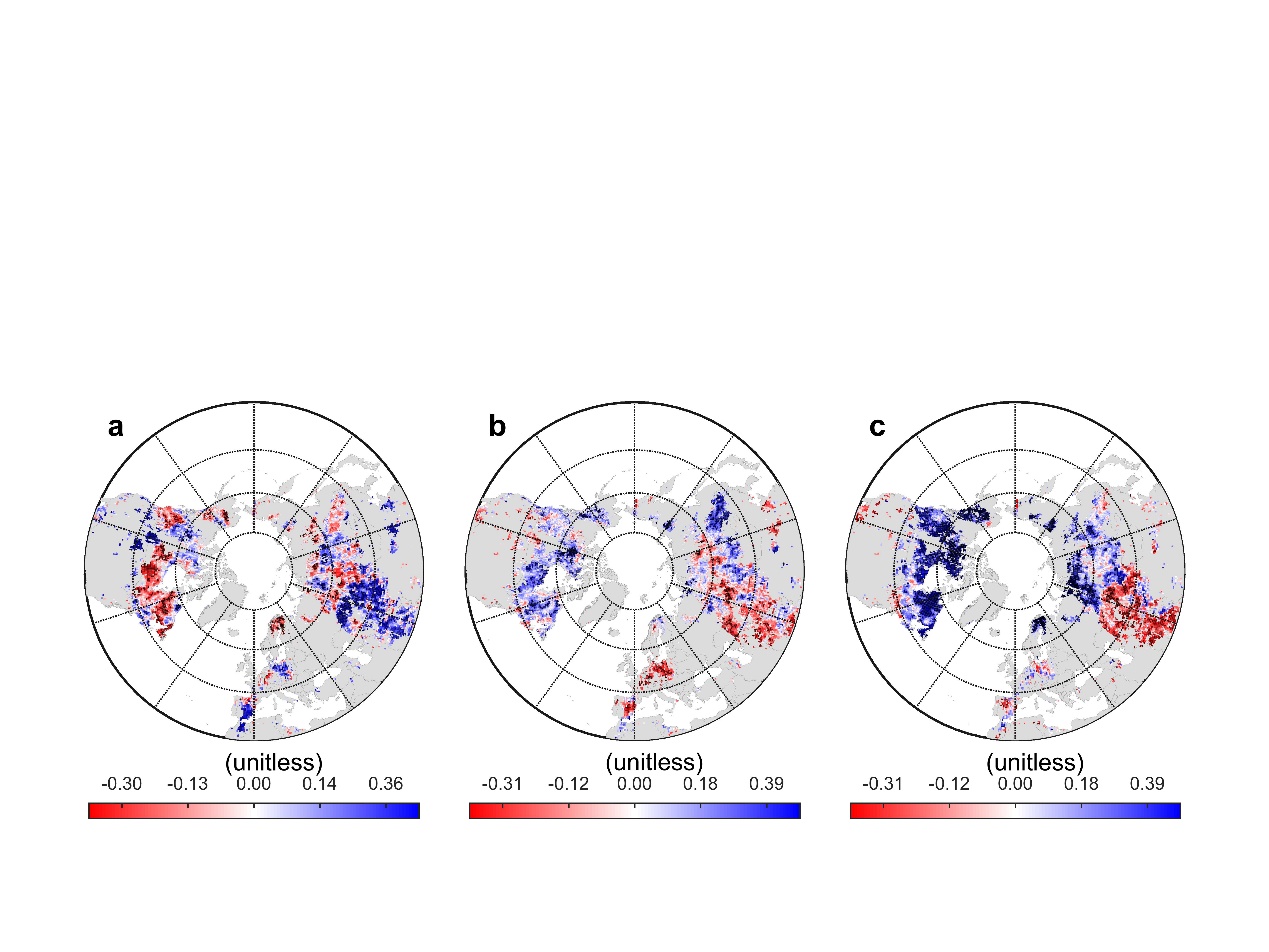


**Supplementary Figure 5. Spatial patterns of the standard regression coefficients between the interannual variations of the mean growing season (April–October) Normalized Difference Vegetation Index (NDVI_GS_) and climate.** A ridge regression was performed between NDVI_GS_ and total growing season precipitation (a), accumulated temperature exposure above (b, TE_H_) and below (c, TE_L_) the 99^th^ percentile of the daily temperature distribution for the growing seasons during the period 1982–2012. Stratified regions are statistically significant at *p* < 0.05. Regions with multi-year mean NDVI values < 0.1 during 1982–2012 were discarded from our analyses (blank regions). **Please note that we defined the extreme high temperature threshold (99^th^) for each pixel based on all daily temperature observations during growing seasons in periods of 1982-2012, thus, such extreme temperature did not occur for many years (that means we can’t calculate the TE_H_, and we thus can’t perform the ridge regression) during 1982-2012 in a large part of study region (i.e., most of blank regions in this figure).**

Supplementary Figure 6.


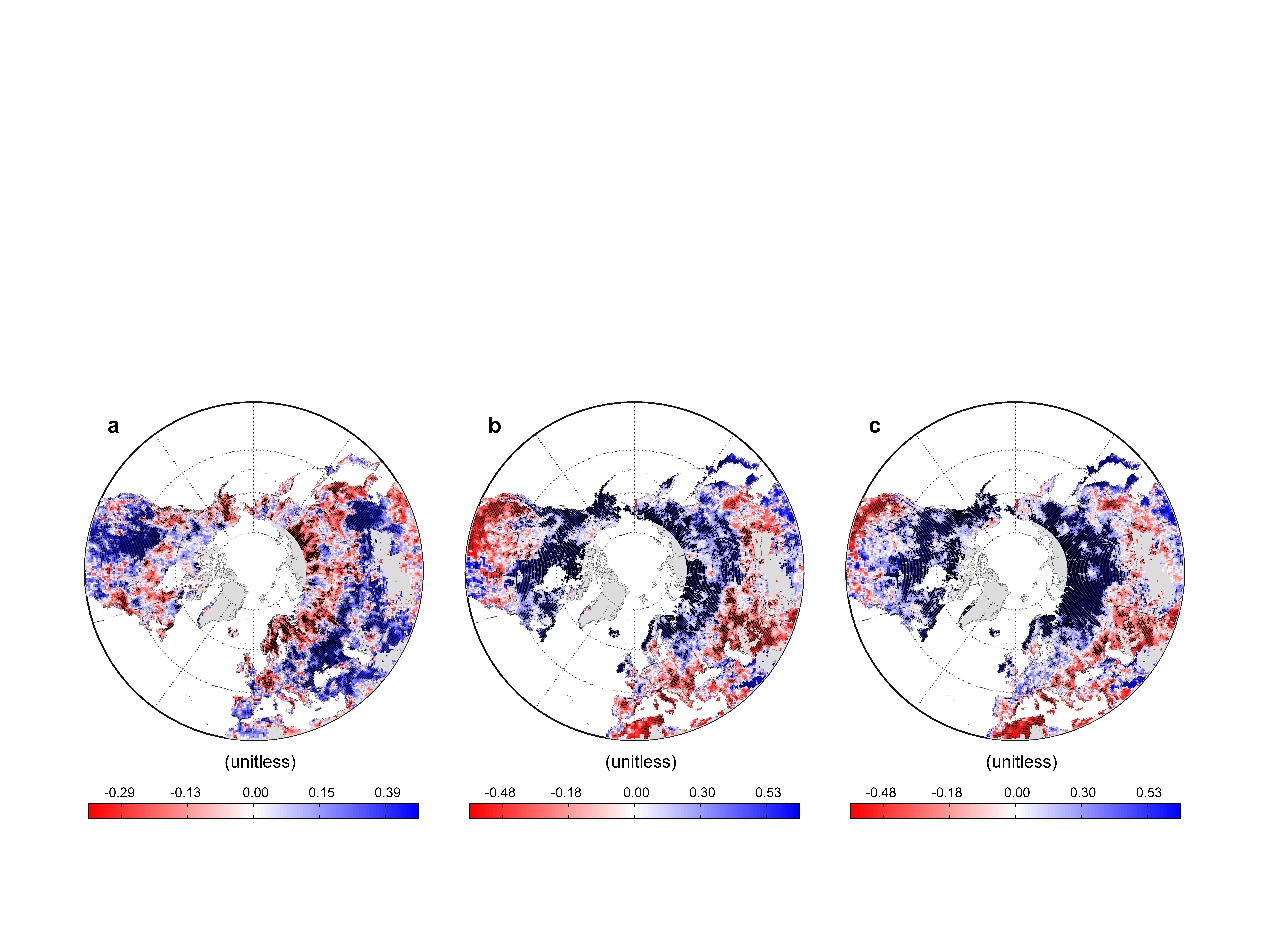


**Supplementary Figure 6. Spatial patterns of the standard regression coefficients between the interannual variations of the mean growing season (April–October) Normalized Difference Vegetation Index (NDVI_GS_) and climate.** Multivariate linear regression was performed between NDVI_GS_ and total growing season precipitation (a), accumulated temperature exposure above (b, TE_H_) and below (c, TE_L_) the 95^th^ percentile of the daily temperature distribution for the growing seasons during the period 1982–2012. Stratified regions are statistically significant at *p* < 0.05. Regions with multi-year mean NDVI values < 0.1 during 1982–2012 were discarded from our analyses (blank regions).

Supplementary Figure 7.


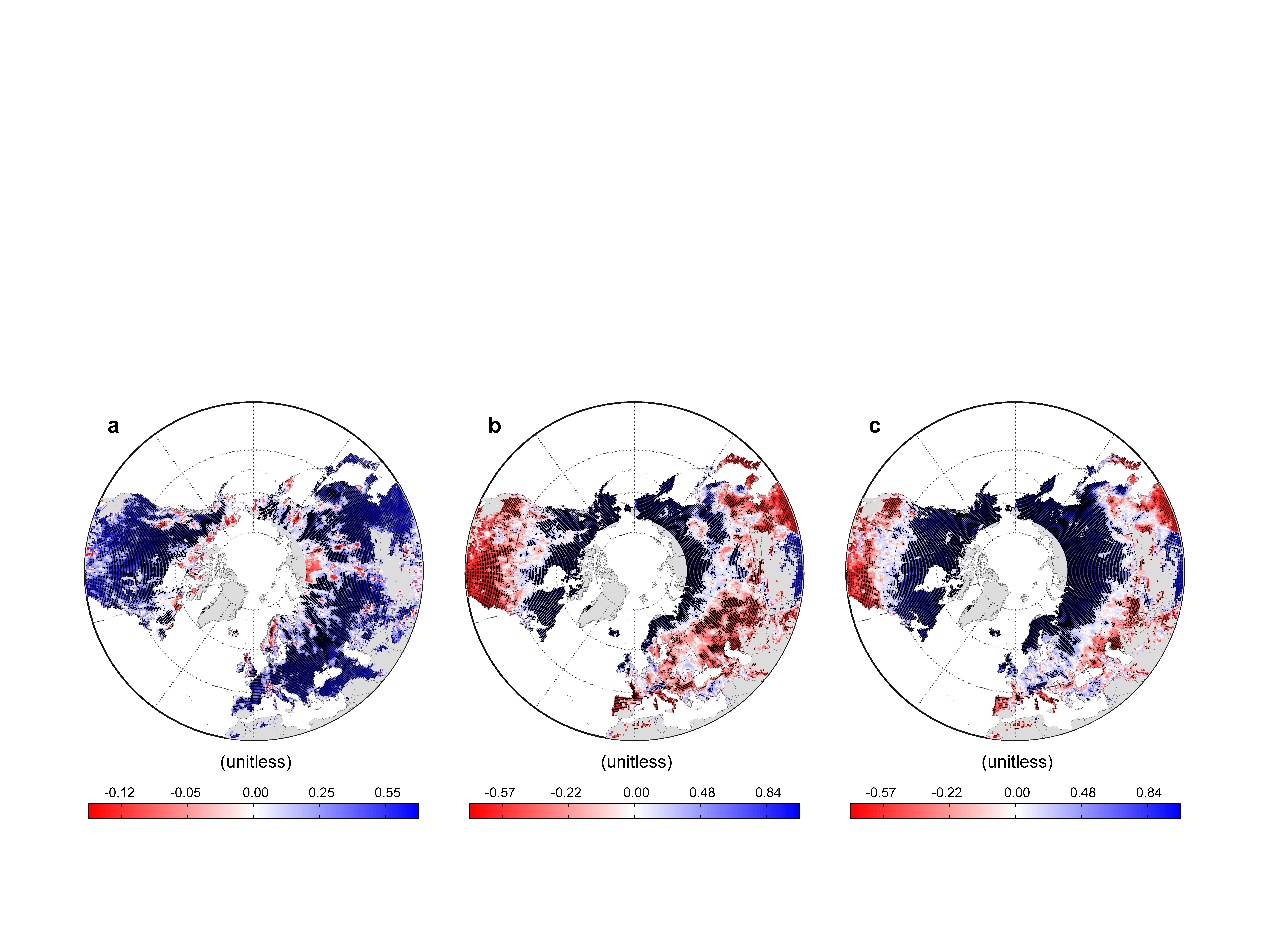


Supplementary Figure 7. Spatial patterns in the standard regression coefficients between the interannual variations of the mean growing-season (April–October) net primary productivity (NPP_GS_) from four land surface models and climates. Ridge regression was performed between NPP_GS_ and total growing-season precipitation (a), accumulated temperature exposure above (b, TE_H_) and below (c, TE_L_) the 95th percentile of the daily temperature distribution for the growing seasons during the period 1982-2010. Stratified regions are statistically significant at *p* < 0.05. Regions with multi-year mean NDVI values < 0.1 during 1982-2012 were discarded from our analyses (blank regions).

Supplementary Figure 8.


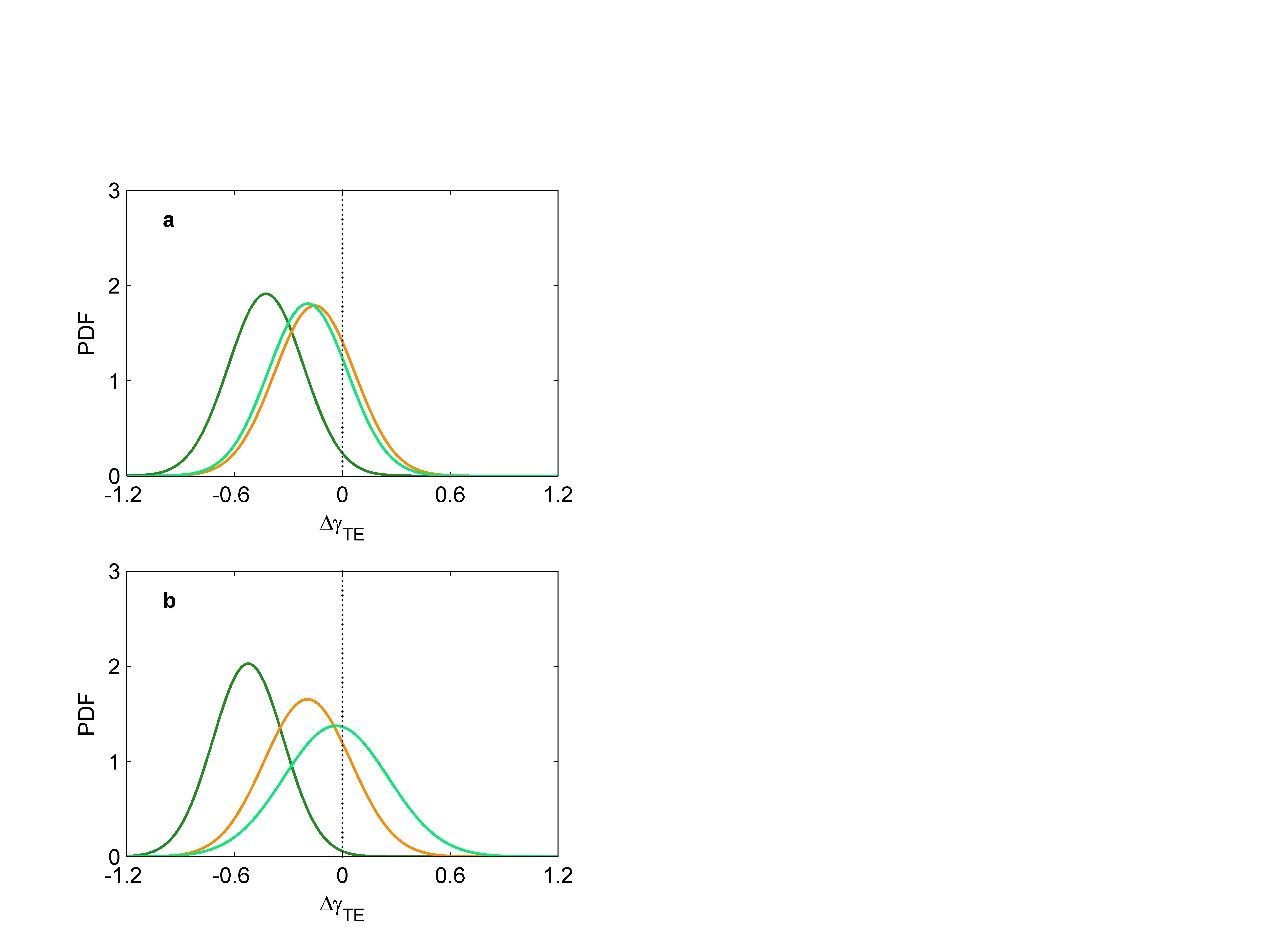


Supplementary Figure 8. Probability density function (PDF) of the differences in the standard regression coefficients between mean growing-season (April–October) net primary productivity (NPP_GS_) from four land surface models and accumulated temperature exposures above (TE_H_) and below (TE_L_) the extremely high temperature threshold. PDF of the differences in the standard regression coefficients between NPP_GS_, and accumulative growing-season TE_H_ and TE_L_ for forest (dark green), shrub (orange), and grass (grass green) in the temperate (a) and the boreal (b) Northern Hemisphere. In this analysis, we only considered pixels with significant (*p* < 0.05) ridge regressions between NPP_GS_, and total growing-season precipitation, and TE_H_ and TE_L_.

Supplementary Figure 9


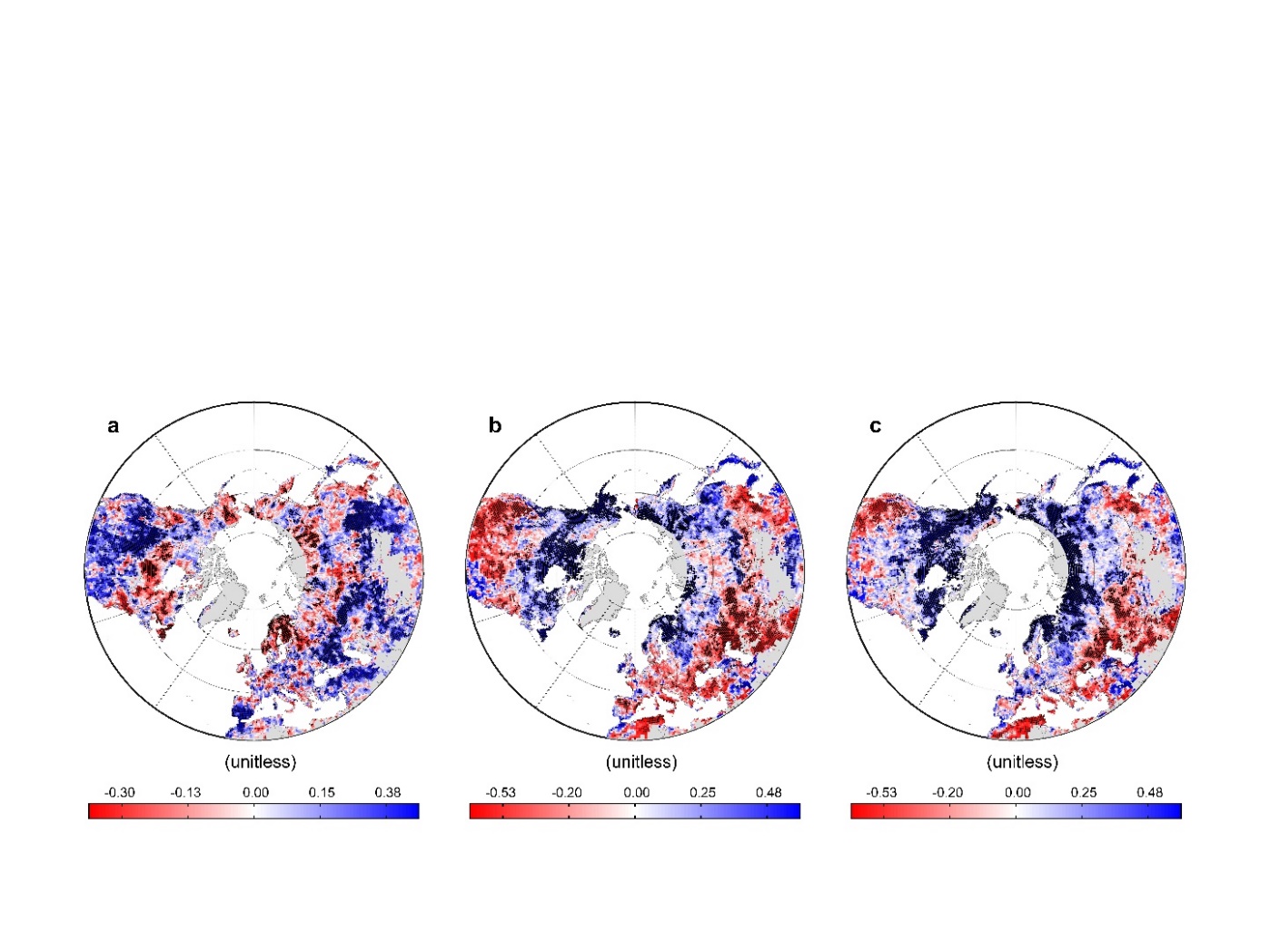


Supplementary Figure 9. Spatial patterns in the standard regression coefficients between the interannual variations of the mean growing-season (May–September) Normalized Difference Vegetation Index (NDVI_GS_) and climates. Ridge regression was performed between NDVI_GS_, and total growing-season precipitation (a), accumulated temperature exposure above (b, TE_H_) and below (c, TE_L_) the 95^th^ percentile of the daily temperature distribution for the growing seasons during the period 1982-2012. Stratified regions are statistically significant at *p* < 0.05. Regions with multi-year mean NDVI values < 0.1 during 1982-2012 were discarded from our analyses (blank regions).

Supplementary Figure 10


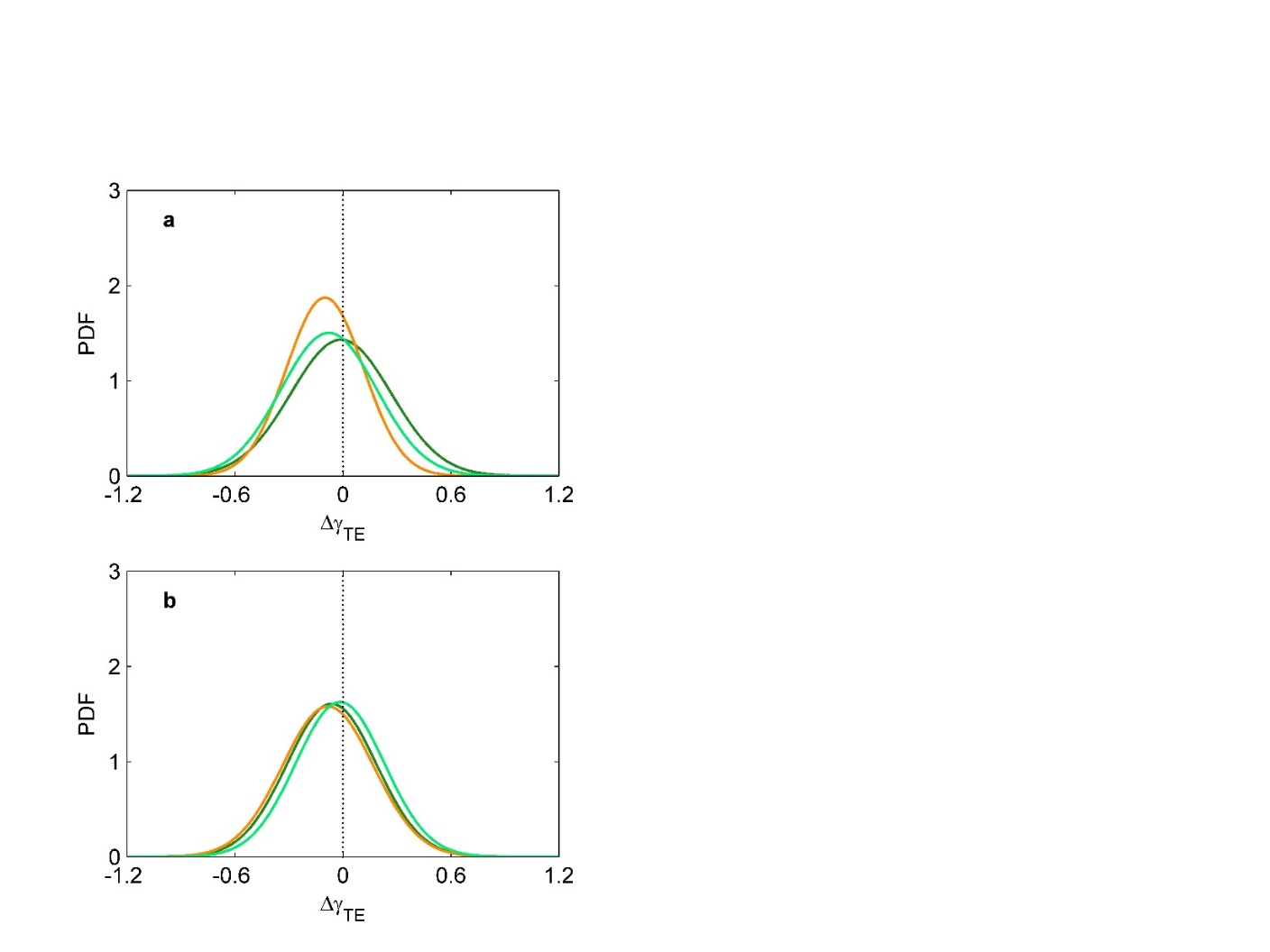


Supplementary Figure 10. Probability density function (PDF) of the differences in the standard regression coefficients between mean growing-season (May-September) Normalized Difference Vegetation Index (NDVI_GS_) and accumulated temperature exposure above (TE_H_) and below (TE_L_) the extremely high temperature threshold. PDF of the differences in the standard regression coefficients between NDVI_GS_, and TE_H_ and TE_L_ for forests (dark green), shrub (orange), and grass (grass green) in the temperate (a) and the boreal (b) Northern Hemisphere. In this analysis, we only considered pixels with significant (*p* < 0.05) ridge regressions between NDVI_GS_, and total growing-season precipitation, and TE_H_ and TE_L_.

Supplementary Figure 11


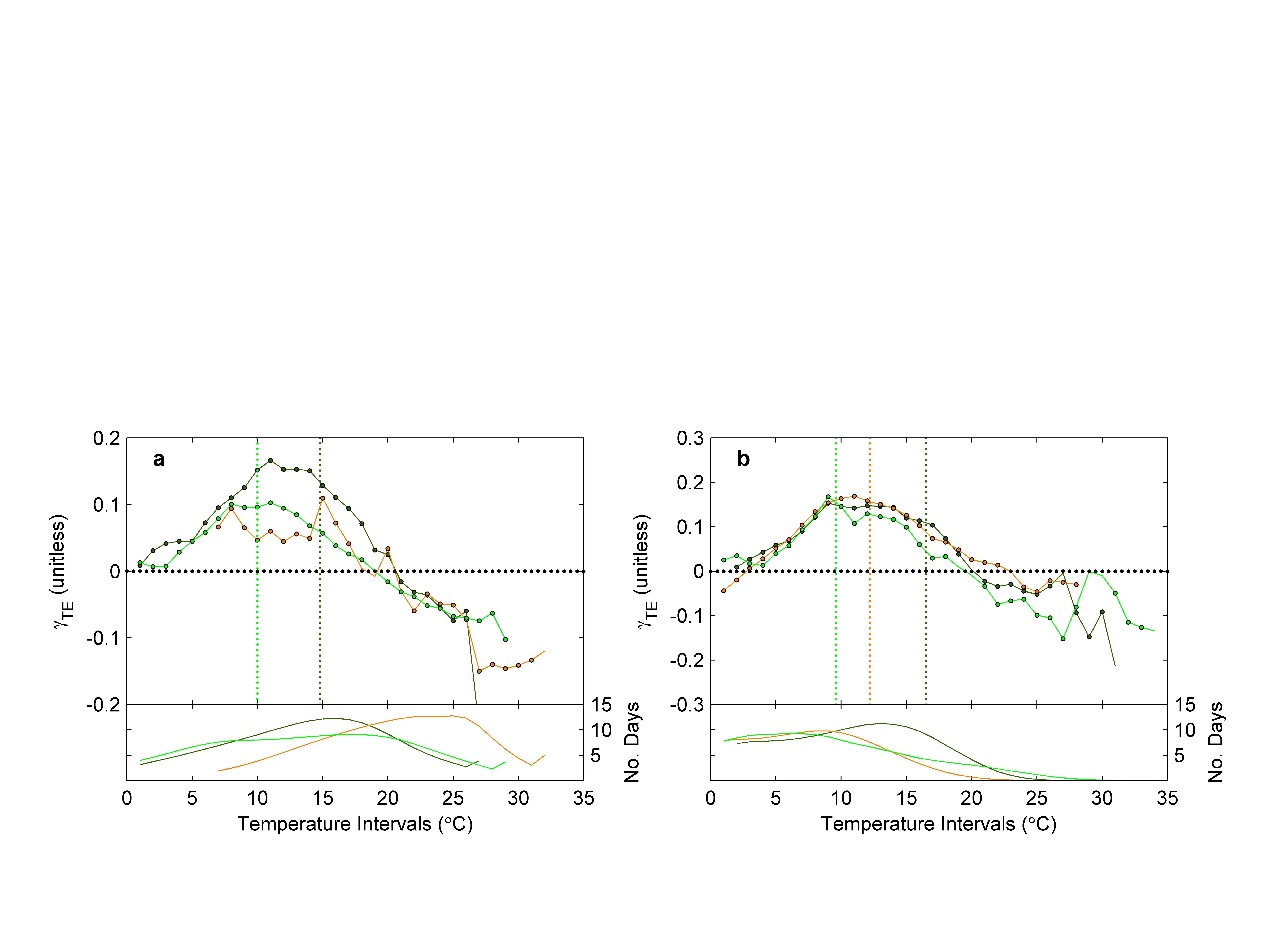


Supplementary Figure 11. Nonlinear relationship between the responses of the mean growing season (April–October) net primary productivity (NPP_GS_) from 4 land surface models to temperature exposures ($\gamma_{TE}$) and TE within different temperature ranges. The nonlinear relationship between $\gamma_{TE}$ of NPP_GS_ and TE in different temperature ranges (with 1 ^o^C intervals) for forest (dark green), shrub (orange), and grass (grass green) in the temperate (a) and the boreal (b) Northern Hemisphere in period of 1982–2010. Graphs at the top of each frame display the changes in the standardized responses of NPP_GS_ in response to one TE within a specific temperature range. The curves are standardized so that the exposure-weighted impact is zero. Markers on lines indicate a significant response of NPP_GS_ to TE within specific temperature ranges. The vertical dashed lines at the top of each frame indicate temperature thresholds for the nonlinear relationship between the $\gamma_{TE}$ of NPP_GS_ and TE for forest (dark green), shrub (orange), and grass (grass green). The average number of days within each 1 ^o^C interval for forest (dark green), shrub (orange), and grass (grass green) during growing seasons between 1982 and 2010 are shown in the bottom part of each frame.

Supplementary Figure 12


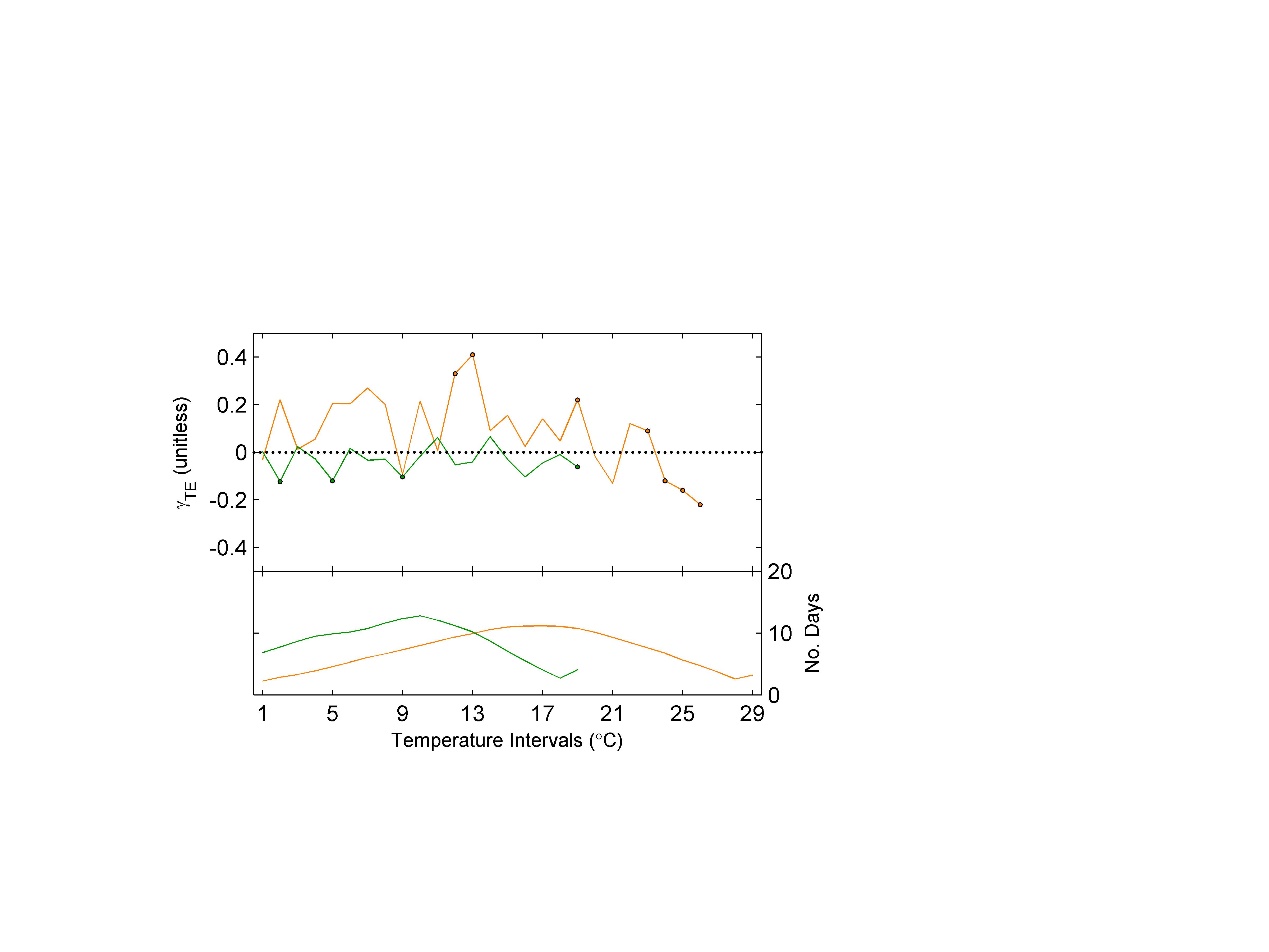


Supplementary Figure 12. Nonlinear patterns in the responses of tree ring index (TRI) to temperature exposures ($\gamma_{TE}$) in the temperate (orange line) and the boreal (green line) Northern Hemisphere (NH) during 1982-2012. Graphs at the top of the frame display changes in $\gamma_{TE}$ of TRI in response to TE within a specific 1 ºC temperature interval. Curves are standardized so that the exposure-weighted impact is zero. Markers on lines indicate significant $\gamma_{TE}$. The average number of days of temperature exposure to each one-degree Celsius interval for forests in the temperate (orange) and the boreal (green line) NH during the growing season is shown at the bottom of the frame.

Supplementary Figure 13


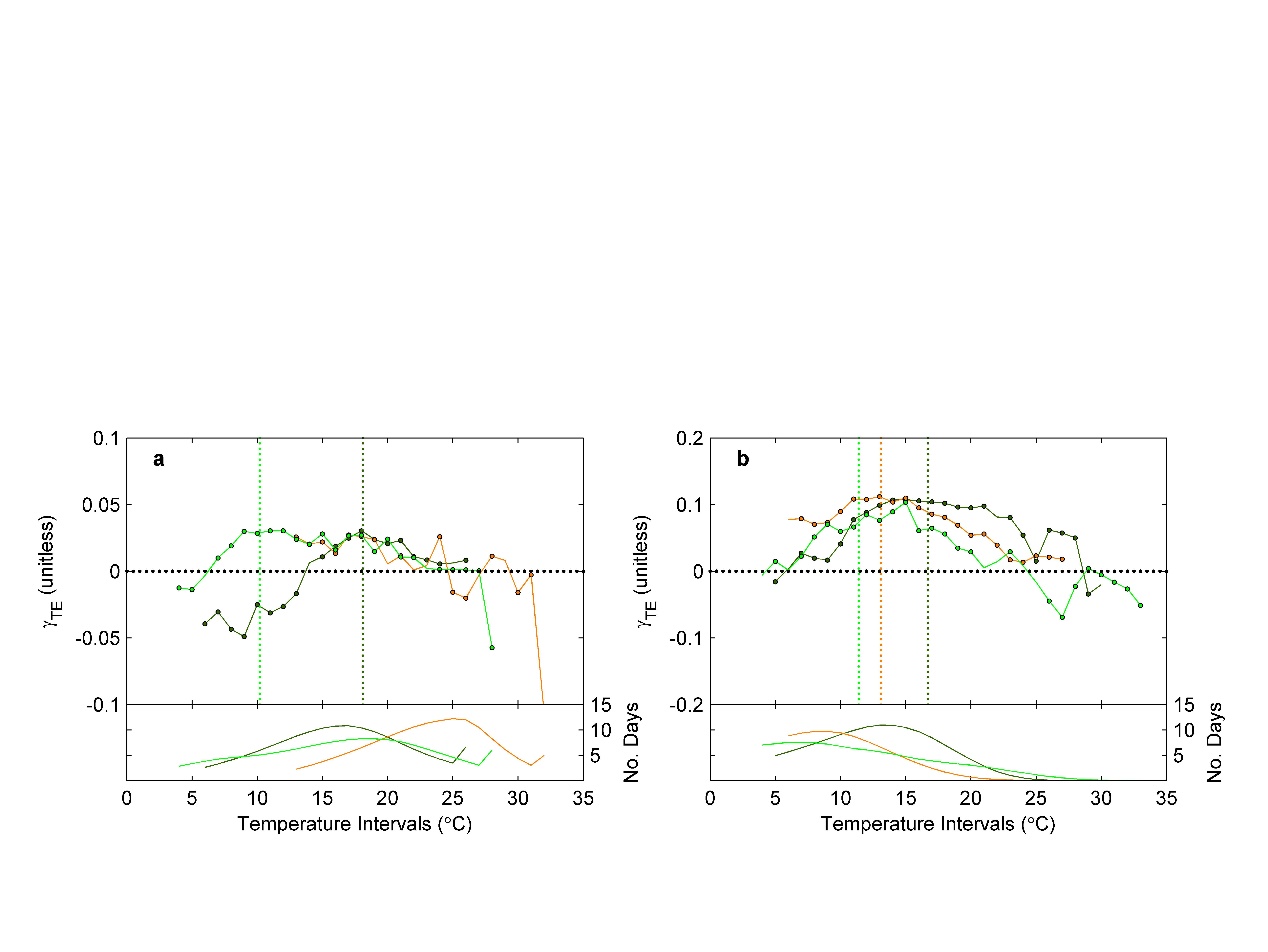


Supplementary Figure 13. Nonlinear relationship between the response coefficients of the mean growing season (May-September) Normalized Difference Vegetation Index (NDVI_GS_) to temperature exposures ($\gamma_{TE}$) and TE within different temperature ranges. The nonlinear relationship between $\gamma_{TE}$ of NDVI_GS_ and TE in different temperature ranges (with 1 ^o^C intervals) for forest (dark green), shrub (orange), and grass (grass green) in the temperate (a) and the boreal (b) Northern Hemisphere for 1982–2012. Graphs at the top of each frame display the changes in the standardized response coefficients of the NDVI_GS_ to one TE within a specific temperature ranges. The curves are standardized so that the exposure-weighted impact is zero. Markers on lines indicate a significant response of NDVI_GS_ to TE. The vertical dashed lines at the top of each frame indicate temperature thresholds for the nonlinear relationship between the $\gamma_{TE}$ of NDVI_GS_ and TE for forest (dark green), shrub (orange), and grass (grass green). The average number of days within each 1 ^o^C interval for forest (dark green), shrub (orange), and grass (grass green) during growing seasons between 1982 and 2012 are shown in the bottom part of each frame. Note that, during the period 1982-2012, temperate shrubland biome shows a consistent decrease in $\gamma_{TE}$in response to TE as the temperature increases.

Supplementary Figure 14.


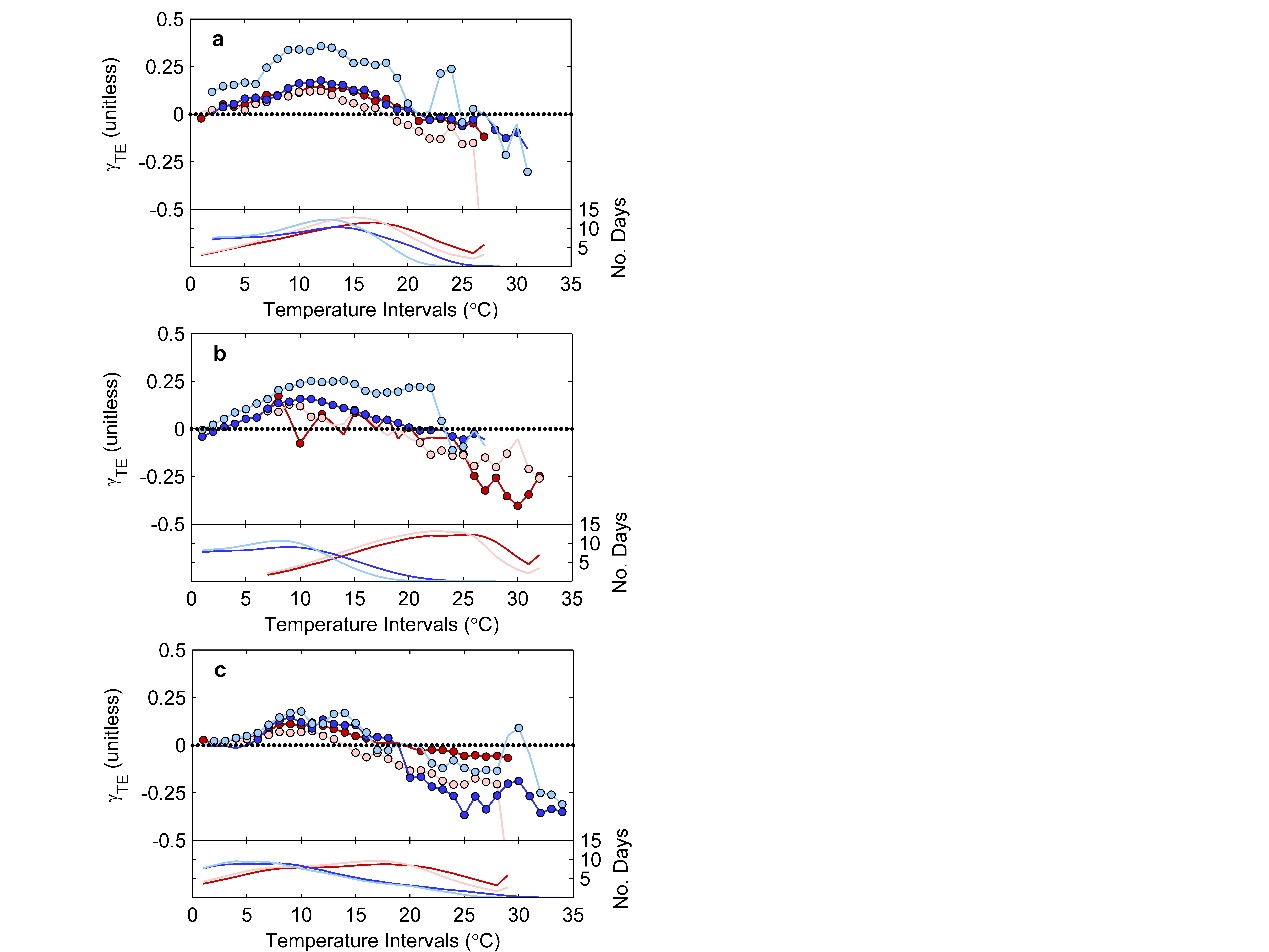


Supplementary Figure 14. Comparison of the nonlinear relationships between the interannual response of the growing-season (April–October) net primary productivity (NPP_GS_) from four land surface models to temperature exposures ($\gamma_{TE}$) and TE within different temperature ranges in years with more and fewer extremely high temperature (EHT) occurrences. The top part of each frame shows the nonlinear relationships between $\gamma_{TE}$ of NPP_GS_ and TE within different temperature ranges in years with more (darker lines) and fewer (lighter lines) EHT occurrences for forests (a), shrublands (b), and grasslands (c) in the temperate (red lines) and the boreal (blue lines) Northern Hemisphere between 1982 and 2010. For each pixel within each biome, we selected 7 years within the period 1982–2010 with more EHT occurrences and 7 years within this period with fewer EHT occurrences compared to the mean number of EHT occurrences over the same period. Lines at the bottom of each frame display the average number of days within different temperature ranges in cases with more (darker lines) and fewer (lighter lines) EHT occurrences in the temperate (red lines) and boreal (blue lines) Northern Hemisphere for each biome.

Supplementary Figure 15


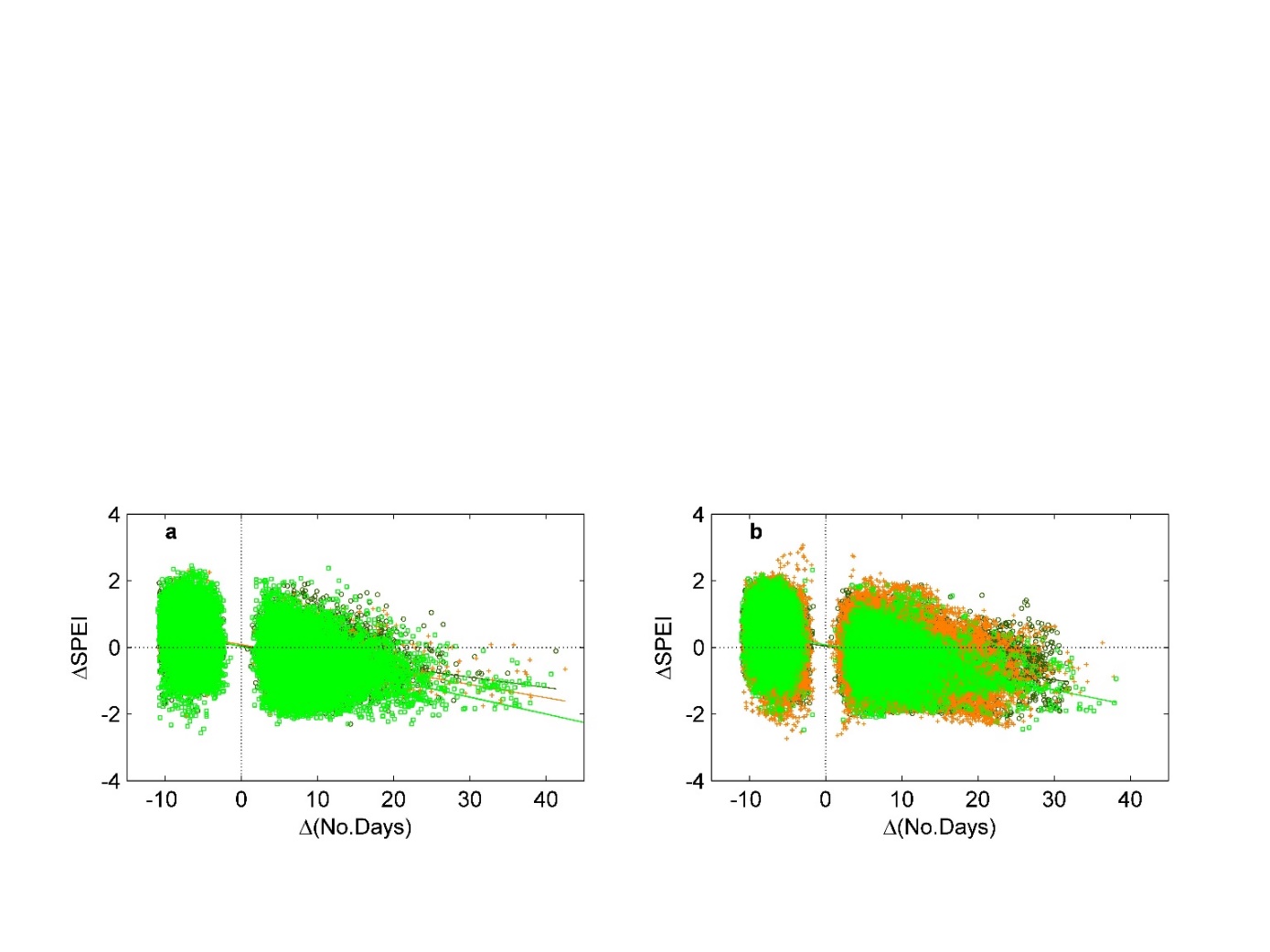


Supplementary Figure 15. Relationships between the difference in Standardized Precipitation-Evapotranspiration Index (*∆SPEI*), and differences in the number of days with extremely high temperature (EHT) occurrences, for different biomes. Scatter plots display the relationships between *∆SPEI* and differences in the number of days with EHT occurrences (defined as exposures to temperatures above the 95^th^ percentile of daily temperature distribution for growing seasons during 1982–2012) under more or fewer^†^ EHT occurrences *vs.* mean EHT occurrences in forests (dark green markers), shrub (orange markers), and grass (grass green markers) in the temperate (a) and the boreal (b) Northern Hemisphere between 1982 and 2012. Solid lines in (a) and (b) indicate the linear fits of the relationships between the *∆SPEI* and the difference in number of days with EHT for the different biomes. All linear fits in (a) and (b) are statistically significant (*p* < 0.05). ^†^We selected 7 years with lower and upper EHT occurrences within the period of 1982–2012 for each pixel of each vegetation type.

Supplementary Figure 16


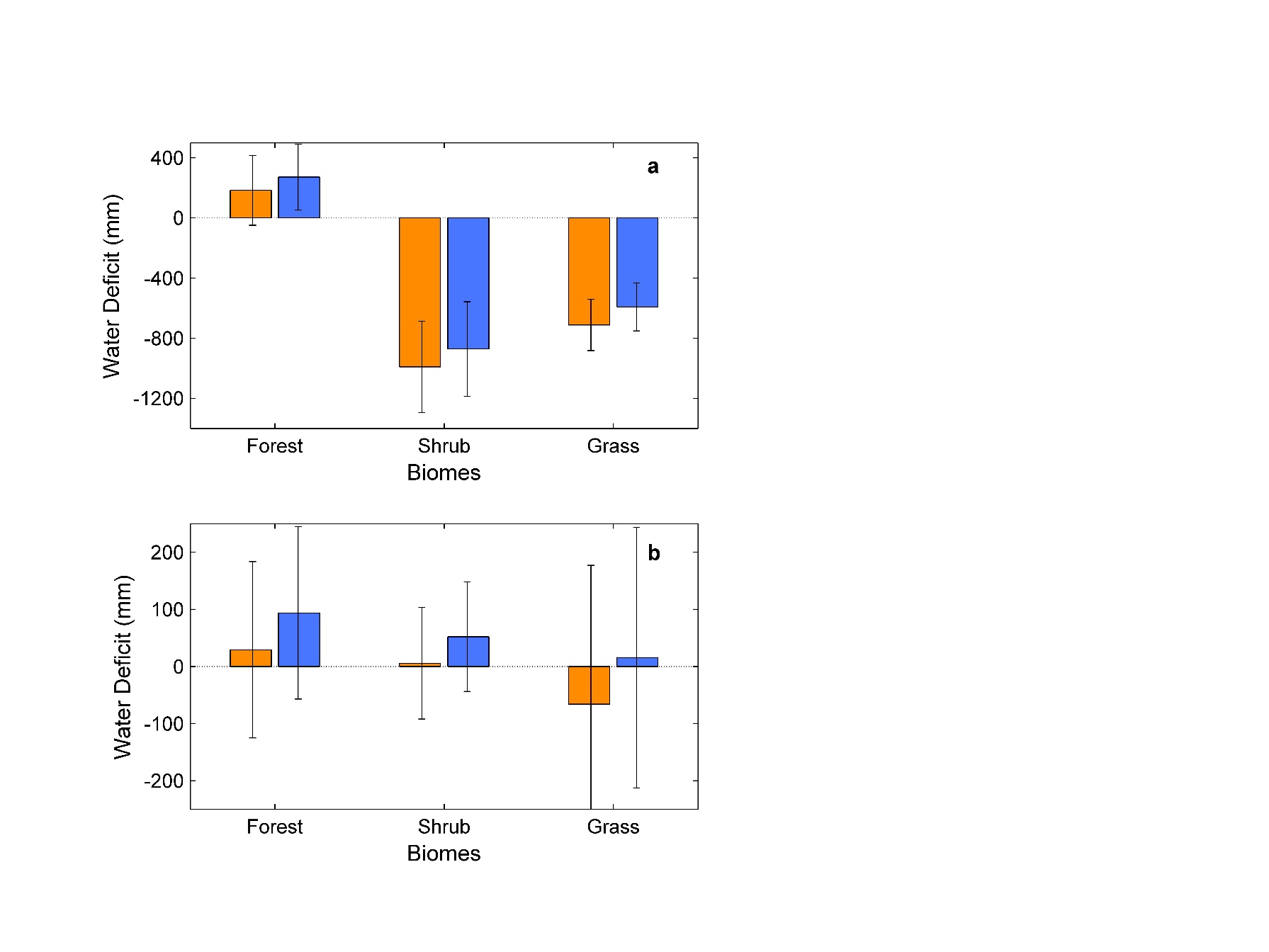


Supplementary Figure 16. Comparisons of mean growing-season (April-October) water deficits between years with more (orange bars) and fewer (blue bars) extremely high temperature events for different vegetation types in temperate (a) and boreal (b) Northern Hemisphere (NH). Differences in mean water deficits between years with more and fewer extremely high temperature events are statistically significant (*p* < 0.05) for all three vegetation types in both the temperate and the boreal NH. Water deficit is calculated here as the difference between total growing-season precipitation and total growing-season potential evapotranspiration.

Supplementary Figure 17





Supplementary Figure 17. Fractions of water taken up by trees, shrubs, and grasses from different soil layers during growing season in temperate Northern Hemisphere. Three different soil layers are roughly defined here, with shallow, middle and deep layer corresponding to 0-20/30cm, 20/30-50/70cm, >50/70cm, respectively. Note that the definitions for shallow, middle and deep layers are quite different among different studies (for details see Supplementary Table 2).

**References**

Liang E, Eckstein D, Liu H (2008) Climate-growth relationships of relict *Pinus tabulaeformis* at the northern limit of its natural distribution in northern China. Journal of Vegetation Science*,* 19, 393-406.

Liang E, Shao X, Liu H, Eckstein D (2007) Tree-ring based PDSI reconstruction since AD 1842 in the Ortindag Sand Land, east Inner Mongolia. Chinese Science Bulletin*,* 52, 2715-2721.

Wu H, Li, X. Y., Jiang, Z., Chen, H., Zhang, C., & Xiao, X. (2016). Contrasting water use pattern of introduced and native plants in an alpine desert ecosystem, Northeast Qinghai–Tibet Plateau, China. Science of the Total, 542, 182-191.

Prieto, I., Pugnaire, F. I., & Ryel, R. J. (2014). Water uptake and redistribution during drought in a semiarid shrub species. Functional Plant Biology, 41, 812-819.

Asbjornsen, H., Mora, G., & Helmers, M. J. (2007). Variation in water uptake dynamics among contrasting agricultural and native plant communities in the Midwestern US. Agriculture, Ecosystems & Environment, 121, 343-356.

Bertrand, G., Masini, J., Goldscheider, N., Meeks, J., Lavastre, V., Celle‐Jeanton, H., & Hunkeler, D. (2014). Determination of spatiotemporal variability of tree water uptake using stable isotopes (δ^18^O, δ^2^H) in an alluvial system supplied by a high‐altitude watershed, Pfyn forest, Switzerland. Ecohydrology, 7, 319-333.

Dai, Y., Zheng, X. J., Tang, L. S., & Li, Y. (2015). Stable oxygen isotopes reveal distinct water use patterns of two *Haloxylon* species in the Gurbantonggut Desert. Plant and Soil, 389, 73-87.

Prechsl, U. E., Burri, S., Gilgen, A. K., Kahmen, A., & Buchmann, N. (2015). No shift to a deeper water uptake depth in response to summer drought of two lowland and sub-alpine C_3_-grasslands in Switzerland. Oecologia, 177, 97-111.

Zheng, X. R., Zhao, G. Q., Li, X. Y., Li, L., Wu, H. W., Zhang, S. Y., & Zhang, Z. H. (2015). Application of stable hydrogen isotope in study of water sources for *Caragana microphylla* bushland in Nei Mongol. Chinese Journal of Plant Ecology, 39, 184-196. (in Chinese with English abstract)

Dai Y, Zheng X J, Tang L S, Li Y. (2014). Dynamics of water usage in *Haloxylon ammodendron* in the southern edge of the Gurbantünggüt Desert. Chinese Journal of Plant Ecology, 38, 1214-1225. (in Chinese with English abstract)

Nippert, J. B., & Knapp, A. K. (2007). Linking water uptake with rooting patterns in grassland species. Oecologia, 153, 261-272.

Liu, S., Chen, Y., Chen, Y., Friedman, J. M., Hati, J. H. A., & Fang, G. (2015). Use of ^2^H and ^18^O stable isotopes to investigate water sources for different ages of *Populus euphratica* along the lower Heihe River. Ecological Research, 30, 581-587.

Jia, Z., Zhu, Y., & Liu, L. (2012). Different water use strategies of juvenile and adult *Caragana intermedia* plantations in the Gonghe Basin, Tibet Plateau. PLoS ONE, 7, e45902, doi:10.1371/journal.pone.0045902.

Li, W., Yan, M., Qingfeng, Z., & Xingchang, Z. (2013). Groundwater use by plants in a semi-arid coal-mining area at the Mu Us Desert frontier. Environmental Earth Sciences, 69, 1015-1024.

1. Loveland T*, et al.* (2000) Development of a global land cover characteristics database and IGBP DISCover from 1 km AVHRR data. *International Journal of Remote Sensing* 21(6-7):1303-1330.
